# A Systematic Review of the Mortality from Untreated Leptospirosis

**DOI:** 10.1371/journal.pntd.0003866

**Published:** 2015-06-25

**Authors:** Andrew J. Taylor, Daniel H. Paris, Paul N. Newton

**Affiliations:** 1 Lao Oxford Mahosot Hospital Wellcome Trust Research Unit (LOMWRU), Microbiology Laboratory, Mahosot Hospital, Vientiane, Laos; 2 Centre for Tropical Medicine and Global Health, Nuffield Department of Clinical Medicine, Churchill Hospital, University of Oxford, Oxford, United Kingdom; 3 Mahidol–Oxford Research Unit (MORU), Faculty of Tropical Medicine, Mahidol University, Bangkok, Thailand; University of California San Diego School of Medicine, UNITED STATES

## Abstract

**Background:**

Leptospirosis occurs worldwide, but the global incidence of human disease and its mortality are not well understood. Many patients are undiagnosed and untreated due to its non-specific symptoms and a lack of access to diagnostics. This study systematically reviews the literature to clarify the mortality from untreated leptospirosis. Results will help quantify the global burden of disease and guide health policies.

**Methodology/Principal Findings:**

A comprehensive literature search was performed to identify untreated patient series. Included patients were symptomatic, but asymptomatic patients and those who had received antibiotics, dialysis or who were treated on Intensive Care Units were excluded. Included patients had a confirmed laboratory diagnosis by culture, PCR, or serological tests. Data was extracted and individual patient series were assessed for bias. Thirty-five studies, comprising 41 patient series and 3,390 patients, were included in the study. A high degree of bias within studies was shown due to limitations in study design, diagnostic tests and missing data. Median series mortality was 2.2% (Range 0.0 – 39.7%), but mortality was high in jaundiced patients (19.1%) (Range 0.0 – 39.7%), those with renal failure 12.1% (Range 0-25.0%) and in patients aged over 60 (60%) (Range 33.3-60%), but low in anicteric patients (0%) (Range 0-1.7%).

**Conclusions:**

This systematic review contributes to our understanding of the mortality of untreated leptospirosis and provides data for the estimation of DALYs attributable to this disease. We show that mortality is significantly higher in older patients with icteric disease or renal failure but is lower in younger, anicteric patients. Increased surveillance and accurate point-of-care diagnostics are required to better understand the incidence and improve diagnosis of disease. Empirical treatment strategies should prioritize early treatment to improve outcomes from leptospirosis.

## Author Summary

Leptospirosis is a common cause of fever in the developing world but often goes undiagnosed and untreated due to its non-specific clinical features and the limited availability of point-of-care diagnostics. This review systematically evaluated available literature to clarify the mortality from untreated leptospirosis. Untreated patients were defined as patients not receiving antibiotics, dialysis, or treatment on an Intensive Care Unit. All patients had a confirmed laboratory diagnosis of leptospirosis through culture, PCR or serological tests. Results showed that mortality from untreated leptospirosis is significant in older patients and those who develop complications such as jaundice and renal failure, but mortality is low in younger patients and those with anicteric disease. There was a high degree of bias within studies due to limitations in diagnostics and missing data. The data presented in this review, when coupled with improved understanding of the true incidence of the disease, will help estimate the burden of disease from leptospirosis. Increased surveillance and accurate point-of-care diagnostics are required to better understand the incidence of disease and outcomes from leptospirosis. Empirical treatment strategies of undifferentiated fever should focus on early treatment of fever to reduce mortality from leptospirosis.

## Introduction

Leptospirosis is a bacterial zoonosis caused by pathogenic *Leptospira* species which are transmitted to humans by exposure to water containing the urine of infected mammals, predominantly rodents [1]. The disease occurs worldwide and over 853,000 cases and 48,000 deaths are estimated to occur each year [2]. Incidence is highest in tropical regions, including the Asia-Pacific, Latin America, and the Caribbean, where there is an estimated incidence of >10 cases per 100,000 population per year [3] Around one billion people are thought to reside in urban slum areas where frequent outbreaks occur following heavy seasonal rains [4], most notably the recent epidemics in Nicaragua in 2007 and the Philippines in 2009 [5]. Despite the wide availability of effective antibiotic treatment, leptospirosis remains under recognized, mainly due to its non-specific clinical manifestations within a wide differential diagnostic spectrum. The current gold standard diagnostic techniques: culture and the microscopic agglutination test (MAT), are expensive, not useful for early diagnosis, require considerable expertise, and are impractical in resource poor settings. To date there is no widely deployable and reliable point-of-care test, meaning a large proportion of patients are never diagnosed or treated [6]. Outbreaks are often confused with viral infections, such as dengue fever, leading to delays in treatment and increased mortality [6–8].

Knowledge of the mortality from untreated leptospirosis is important for our understanding of the global burden of disease and the calculation of Disability Adjusted Life Years (DALYs), and will inform empirical fever treatment strategies and economic analyses [9]. The DALYs from leptospirosis are currently unknown and our understanding of the untreated mortality from leptospirosis remains limited. Mortality is thought to depend on host factors such as age, and bacterial factors such as serotype or inoculum size [4,6] but current estimates of mortality vary widely according to the clinical presentation, from 0% in patients with non-severe disease [10] to over 50% in those with Severe Pulmonary Haemorrhagic Syndrome (SPHS) [4,11]. This review aims to improve these estimates and better define the untreated mortality from leptospirosis, based on a comprehensive systematic review of previously published literature.

## Methods

### Eligibility criteria

This review included all studies that contained untreated patients with leptospirosis. Patients were defined as untreated if they had received no leptospirosis-effective antibiotic treatment, were not treated with convalescent serum or admitted to an Intensive Care Unit (ICU), and did not receive dialysis. Included patients were of all ages and presented with symptoms consistent with leptospirosis and a confirmed diagnosis through either identification of *leptospira* by culture or direct microscopy, diagnosis by Polymerase Chain reaction (PCR) or serology through MAT. Patients admitted to hospital for supportive treatment (including IV fluids) were included in the analysis. All study designs and articles in all languages were included in the search. Studies were excluded if the diagnosis was on a clinical basis alone, there was no confirmed laboratory diagnosis for all patients in a clinical series, or if patients had asymptomatic infection. Studies with fewer than 10 patients were excluded to reduce patient selection bias. Inclusion and exclusion criteria are summarized in S1 Table. The primary outcome of the analysis was mortality from leptospirosis. Secondary outcomes were total days of fever, clinical signs and symptoms, and laboratory results for liver and kidney function where available. This review followed the PRISMA statement for systematic reviews (S1 Checklist).

### Information sources and search criteria and study selection

Studies were identified through electronic resources, by scanning reference lists of relevant articles, and from library index catalogues, resulting in a comprehensive collection of published and peer reviewed full text articles. The electronic search was performed using Ovid MEDLINE (1946–Present), Embase Classic (1947–Present), and Global Health (1910–Present) on 28^th^ July 2014 (S1–S3 Figs) and results reviewed manually. The search term used were: “Leptospirosis or leptospira or leptospir*; Weil's disease; Weil’s Syndrome; Swamp Fever; Mud fever; Autumn fever; Akiyami disease; Swineherds disease; Rice field fever; Cane cutters fever; Haemorrhagic Jaundice; Stuttgart disease; Canicola fever; Fort Bragg fever; icterohemorrhagic fever; seven day fever; dairy farm fever” and “Mortality or death”. Authors were not contacted regarding further information, no unpublished or grey literature was obtained, and studies were excluded if the full text was not available. Duplicate articles were removed using the reference manager “Mendeley” (2008–14 Mendeley Ltd, Version 1.12.1). One author (AT) reviewed the title and abstract, and papers were excluded if they did not fit the eligibility criteria. If there was doubt as to whether a paper was appropriate for inclusion then the whole paper was acquired and reviewed for eligibility.

### Summary measures and planned method of analysis and data extraction

One author (AT) extracted all available data and used Google Translate to translate non-English articles. Several articles contained more than one patient series, which were extracted separately, and all series were reviewed to prevent duplication of patient series. Treated patient subgroups were separated from untreated patient subgroups and excluded, and the whole patient series was excluded if separate outcomes were not clearly defined. There is no standardised method for assessing bias in non-interventional observational studies, and an existing data extraction sheet [12] was modified to create standardized criteria to assess bias within each study. Bias was graded according to patient selection and study design, diagnostic criteria, missing data and missing outcomes (S2 Table). To grade diagnostic certainty, we adapted diagnostic criteria from the WHO [13] and a grading system from Phommasone et al. [14]. Grade I diagnosis was identification of spirochetes through microscopy or culture, PCR, or a 4-fold rise in MAT titre, Grade II a single high MAT titre of ≥1:400 in an endemic region or ≥1:100 in a non-endemic region, and Grade III a single high titre MAT with no specified titre or a confirmed diagnosis but no record of the diagnostic method. Due to differences in methodology, inclusion criteria, missing data, and bias across patient series, a statistical meta-analysis was not performed. Each patient series was defined as a separate population and the median and range were used to summarize outcomes across patient series. The primary outcomes of the review was measured as the median mortality across all patient series and termed the “median series mortality”. Secondary outcomes were measured as the median value across patient series. For graphs, 95% Confidence Intervals (CI) of mortality were estimated using the Wilson score method [15]. Data was mapped using an image from NASA—Visible Earth.

## Results

**Fig 1:**
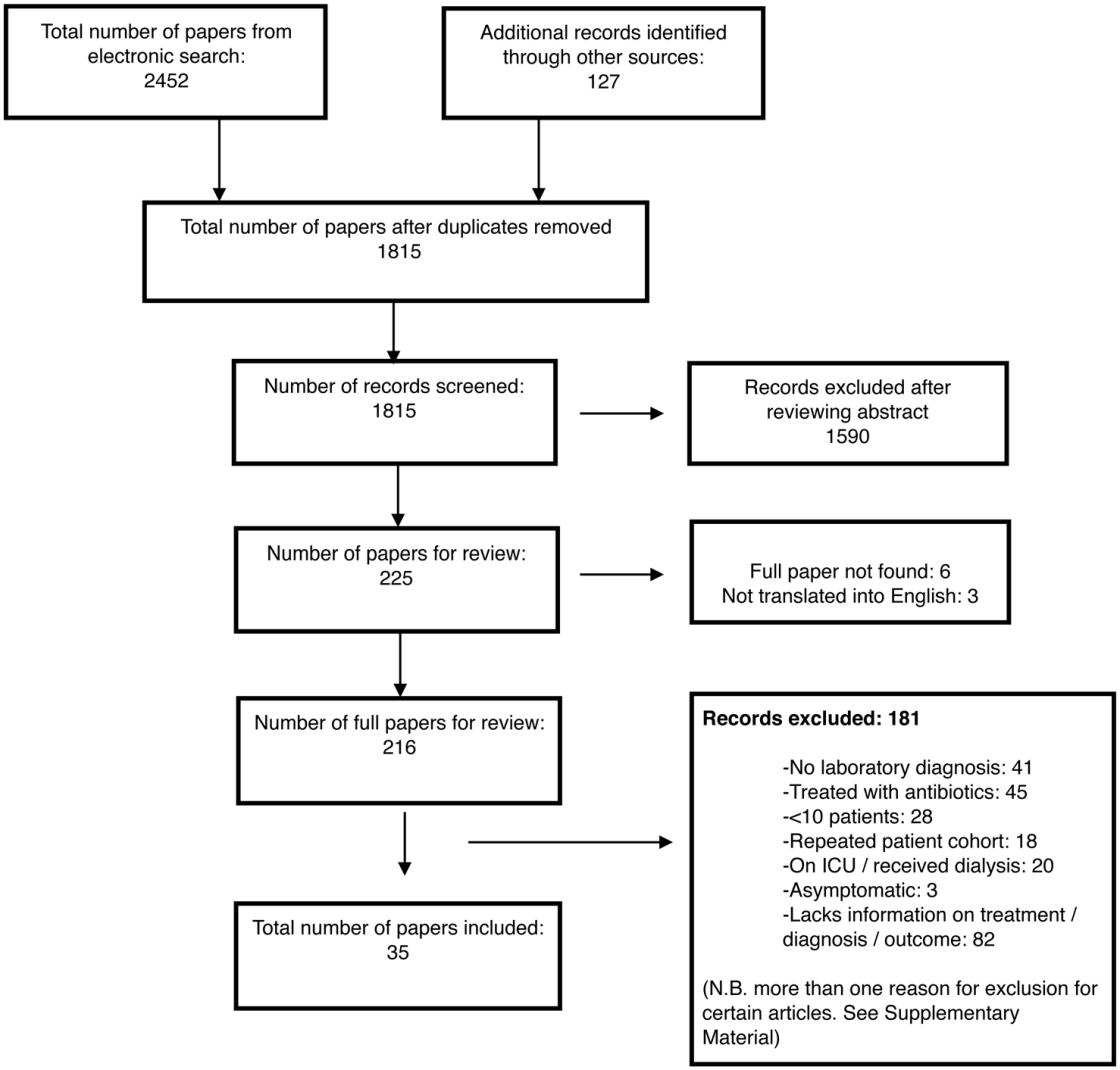
Flow diagram for selection of studies included in the review.

A total of 35 studies, comprising 41 patient series, and containing a total of 4,247 patients were identified for inclusion in the review (Table 1). Within the included patient series, 857 patients had an unknown outcome, were treated, or had no laboratory diagnosis and were excluded (Table 1) and 3,390 patients were included in the final review. Six studies were excluded as the full article was not obtained and 3 excluded as the article could not be translated into English (S3 Table). Details of excluded articles with reasons for exclusion are displayed in Fig 1, with further information for each excluded article given in S4 Table.

**Table 1:** Characteristics of included studies, arranged alphabetically by first author.

Study Title (Reference) (Language if not English)	County, Year of Study	Study design	Untreated Patients and serovar	Median (Unless stated) age (range)	% Male	Median (Unless stated) Duration of Fever (Days) (Range)	Diagnostic test	Proportion of patients jaundiced (%)	Proportion of patients impaired renal function (%)	Mortality (%) (Deaths / Number of patients)	Patients excluded
Baermann G & Smits E. Zentralbl Bakteriol. 1928 [16] (German)	Sumatra, Indonesia 1923–1927	Prospective case series	196 (196/346) *	-	100% (101/101)	-	(196/196) Blood culture or guinea pig inoculation	17% (NA/196)	-	1% (NA/196)	150 patients excluded as no bacteriological confirmation of diagnosis
Berman SJ, et al. Ann Intern Med. 1973 [17]	Vietnam 1971–2	Prospective case series	101 (101/200) *	-	-	8.8 (mean)	(200/200) Single high titre MAT (≥1:400). (27/200) Positive cultures	1.5% (2/150)	26% (22/84) urea>10.7mmol/L	0% (0/101)	49 treated patients and 50 patients with incomplete records excluded
Borg-Petersen C. P Roy Soc Med. 1949 [18]	Denmark 1934–48	Danish reference laboratory. Retrospective case series	254 (254/254) Icterohaemorrhagiae	-	86% (218/254)	-	(254/254) Single high titre MAT (Titre not specified)	65.4% (NA/254)	-	14.6% (37/254)	No patients excluded
	Denmark 1934–48	Danish reference laboratory. Retrospective case series	459 (459/459) Serjo & others	-	68.4% (314/459)	-	(459/459) Single high titre MAT (Titre not specified)	14.7% (NA/459)	-	1.3% (6/459)	No patients excluded
	Denmark 1934–48	Danish reference laboratory. Retrospective case series	95 (95/95) Canicola	-	57% (54/95)	-	(95/95) Single high titre MAT (Titre not specified)	13% (NA/95)	-	0% (0/95)	No patients excluded
Broom JC & Alston JM. Lancet. 1948 [19]	UK 1940–46	UK reference laboratory. Retrospective case series	114 (114/195) Icterohaemorrhagiae	34.4 (100/195)	96.6% (181/189)	-	(114/114) Single high titre (≥1:300)	89% (107/120)	-	21.9% (25/114)	(81/195) patients with no information on mortality excluded
Broom JC. Brit Med J. 1951 [20]	UK 1947–50	UK reference laboratory. Retrospective case series	259 (259/465) Icterohaemorrhagiae	36.7 (361/465)	95% (439/465)	-	(259/259) Single high titre MAT (Titre not specified)	74% (344/465)	-	13.9% (36/259)	(206/465) Patients treated with penicillin excluded
	UK 1947–50	UK reference laboratory. Retrospective case series	70 (70/70) Canicola	36.7 (64/70)	63% (44/70)	-	(70/70) Single high titre MAT (Titre not specified)	17% (10/54)	-	1.4% (1/70)	No patients excluded
Btesh S. T Roy Soc Trop Med H. 1947 [21]	Palestine / Israel 1947	Summary of case reports	15 (15/17) Bovis	41.25 (23–60) (16/17)	100% (17/17)	11 (9–45) (15/15)	(3/15) Autopsy. (12/15) Single high MAT (≥1:280)	100% (17/17)	88% (15/17) “urea raised”	20% (3/15)	2 treated patients excluded
Bulmer E. Brit Med J. 1945 [22]	Normandy, France 1944	NRCT	23 (23/39) *	-	100% (23/23)	-	(NA/29) Single high titre MAT for majority (Titre not specified). (NA/29) Isolation from blood or urine	95% (37/39)	-	8.7% (2/23)	Penicillin treated patients excluded (16/39)
Cavigneaux, et al. Arch Mal Prof Med. Trav Soc Sec. 1948 [23] (French)	Paris, France 1948	Retrospective case series	32 (32/32) Icterohaemorrhagiae	38.25 (17–61) (32/32)	96.6% (28/29)	-	(21/32) “Definitive” Single high titre MAT. (7/32) “Borderline” Single High Titre MAT. (4/32) Autopsy	84.4% (27/32)	-	12.5% (4/32)	No patients excluded
Fairburn AC & Semple SJG. Lancet. 1956 [24]	Kuala Lumpur, Malaysia 1955	RCT	31 (31/83) *	21(mean) (18–35) (31/31)	100% (83/83)	9.4 (Mean) (31/31)	(31/31) Paired serum MAT	6% (2/31)	33% (4/12) urea >14.2mmol/L	0% (0/31)	(52/83) Penicillin and chloramphenicol treated patients excluded
Fletcher W. T Roy Soc Trop Med H.1927 [25]	Malaysia 1925–6	Prospective case series	32 (32/32) *	-	-	8.5 (6–12) (26/32)	(13/20) guinea pig inoculation. (13/21) Positive urine microscopy. (18/21) Positive blood culture	22.8% (7/32)	-	3.1% (1/32)	No patients excluded
Gardner AD & Wylie JAH. Lancet. 1946 [26]	England, UK 1940–45	Retrospective case series. Reference Laboratory	182 (182/182) Icterohaemorrhagiae	-	93.6% (147/157)	-	(182/182) Single high titre MAT (≥1:400)	-	-	8.8% (16/182)	No patients excluded
Hall HE, et al. Ann Intern Med. 1951 [27]	Puerto Rico 1950	NRCT	12 (12/79) *	-	-	8.6 (Mean) (12/12)	(9/12) Guinea pig inoculation. (3/12) paired MAT	0% (0/12)	0% (0/12)	0% (0/12)	(67/79) Treated patients excluded
Hamilton Fairley N. Brit Med J. 1934 [28]	London, UK 1933–4	Retrospective case series	10 (10/10) *	31 (22–60) (9/10)	100% (10/10)	-	(8/10) Single high titre MAT. (≥1:300 for 7/8). (2/10) Autopsy	100% (10/10)	-	20% (2/10)	No patients excluded
Ido Y, et al. J Exp Med. 1918 [29]	Japan 1917	Prospective, non-consecutive case series	20 (20/23) hebdomidis	-	-	-	(20/23) Guinea pig inoculation	0% (0/20)	-	0% (0/20)	Three patients excluded as no confirmed diagnosis
Kocen RS. Brit Med J. 1962 [30]	Malaysia 1959–60	NRCT	33 (33/61) *	-	100% (33/33)	8.25 (mean) (33/33)	(NA/33) “Confirmed by blood culture or serology”	15% (5/33)	-	0% (0/33)	(28/61) Penicillin cohort excluded
Kouwenaar W. Trans Far-East Ass Trop Med. 1925 [31]	Sumatra, Indonesia 1920s	Prospective case series	32 (32/32) *	-	-	-	(27/32) Direct identification of leptospires. (10/32) Demonstration of leptospires on autopsy	100% (32/32)	68% (15/22) “raised urea”	18.8% (6/32)	No patients excluded
	Sumatra, Indonesia 1920s	Prospective case series	58 (58/58) *	-	-	-	(40/58) Direct visualisation in urine. (18/58) Blood culture	0% (58/58)	44.8% (13/29) “raised urea”	1.7% (1/58)	No patients excluded
Kristensen B. Ugeskrift Laeger. 1935 [32] (Danish)	Denmark 1933–5	Retrospective case series	19 (19/19) Icterohaemorrhagiae	40 (13–65) (19/19)	68% (13/19)	-	(19/19) Single High Titre (Titre not specified)	100% (18/18)	-	26.3% (5/19)	No patients excluded
McClain BL, et al. Ann Intern Med. 1984 [33]	Panama 1983	RCT	15 (15/69) *	-	100% (15/15)	7.7 (Mean) (S.D. 1.5) (15/15)	(28/69) Culture positive. (1/29) Serological conversion only	0% (15/15)	-	0% (0/15)	(54/69) Patients treated or with life threatening illness excluded
Minkenhof J. Lancet. 1947 [34]	Netherlands 1946	Retrospective case series	17 (17/49) *	-	-	(2–48) (17/17)	Positive agglutination ≥1:1000 (17/17), (3/17) direct identification of leptospires	0% (0/17)	-	0% (0/17)	(32/49) No laboratory diagnosis
Molner JG, et al. JAMA. 1948 [35]	USA, Detroit 1937–48	Retrospective cases series	78 (78/78) *	40 (3–64) (78/78)	93.6% (73/78)	-	(73/78) Single MAT (≥1:300). (5/78) by autopsy	100% (78/78)	-	39.7% (31/78)	No patients excluded
	USA 1905–1941	Summary of case reports	178 (178/178) *	-	-	-	(178/178) “Confirmed diagnosis”	-	-	24.8% (44/178)	No patients excluded
Mulder J, et al. Geneesk Tijdschr Ned-indië. 1931 [36] [Dutch]	Borneo 1929–30	Prospective case series	50 (50/50) *	“20–50 years old”	-	-	(41/50) Positive guinea pig inoculation. (18/50) MAT Single titre (≥1:400)	8% (4/50)	-	4% (2/50)	No patients excluded
Patterson HM. JAMA. 1947 [37]	Hawaii, USA 1941–1946	Retrospective case series	(44/61) Icterohaemorrhagiae	-	-	15.3 (mean) (44/44)	(44/44) Single high titre MAT (≥1:300). (?/44) Paired and rising titre.	-	-	0% (0/44)	(17/61) Patients received treatment with antibiotics or serum
Robinson CR & Kennedy HF. J R Army Med Corps. 1956 [38]	Malaysia 1953	Retrospective case series	(29/31) *	-	100% (29/29)	- (1–11) (29/29)	(20/23) Positive culture. (28/29) Single high titre MAT. (Titre not specified)	0% (0/29)	58.6% (17/29) >16mg/100 ml blood	0% (0/29)	(2/31) Patients excluded as no laboratory diagnosis
Ross Russell RW. Lancet. 1958 [39]	Malaysia 1957–8	RCT	25 (25/52) *	21 (mean) (18–36) (25/25)	100% (25/25)	9.4 (25/25)	(NA/25) “Culture or serology”	20% (5/25) jaundice. 52%	(13/25) Urea>16mmol/L	0% (0/25)	(27/52) Tetracycline treated patients excluded
Rugiero HR et al. Rev Med Cienc Af. 1948 [40] (Spanish)	Argentina 1948	Retrospective case series	12 (12/12) *	14.5 (10–28) (12/12)	-	-	(12/12) Single high titre MAT (≥1:50)	0% (0/12) jaundice-		0% (0/12)	No patients excluded
Schüffner W. Deut Med Wochenschr. 1941 [41] (German)	Netherlands 1924–39	Retrospective case series. Dutch reference laboratory	272 (272/272) *	-	-	-	(272/272) Single high titre MAT. (Titre not specified)	100% (272/272)	35.8% (38/106) “oliguria”	19.1% (52/272)	No patients excluded
	Netherlands 1924–39	Retrospective case series. Dutch reference laboratory	158 (158/158) *	-	-	-	(158/158) Single high titre MAT. (Titre not specified)	0% (158/158)	5.3% (5/95) “oliguria”	0% (0/158)	No patients excluded
Senekjie HA. JAMA. 1944 [42]	Louisiana, USA 1939–44	Retrospective case series	30 (30/30) *	- (14–68)	96.7% (29/30)	8–37 (30/30)	(24/30) single high titre MAT (≥1:300). (7/30) through direct identification of leptospires	93% (28/30)	100% (30/30) urea >17mmol/L	16.7% (5/30)	No patients excluded
Slot G, Van der Walle N. Geneesk Tijdschr Ned-indië. 1932 [43] [Dutch]	Sumatra, Indonesia 1929–30	Retrospective case series	17 (17/17) *	-	-	(2–15) (17/17)	(3/17) Leptospirosis Culture. (7/17) Animal inoculation. (7/17) Positive agglutination (≥1:400)	47.1% (8/17)	-	0% (0/17)	No patients excluded
Smith J. Brit J Ind Med. 1949 [44]	Scotland, UK 1934–48	Reference laboratory Scotland. Retrospective case series	198 (198/214) *	25.5 (214/214)	52.8% (113/214)	-	(214/214) high titre (≥1:10). (93/187) Animal inoculation	64% (137/214)	59.5% (127/214) urea >14.3mmol/L	7.6% (15/198)	Treated cohort excluded (16/214)
Swan WGA & McKeon JA. Newcastle M J. 1938 [45]	Newcastle, England 1933–7	Retrospective case series	18 (18/30) *	36 (29/30)	100% (30/30)	-	(8/30) Inoculation of Guinea Pig. (9/30) Diagnosed at autopsy. (23/30) Single high titre MAT. (≥1:100 (up to 3 years later))	96.7% (29/30)	100% (16/16) urea >11mmol/L	22.2% (4/18)	(12/30) Treated (serum) cohort excluded
Taylor J & Goyle AM. Indian J Med Res 1931 [46]	Andaman Islands, India 1929	Prospective case series	46 (46/64) *	-	100% (46/46)	7 days (2–16)	(36/64) Blood culture positive. (15/48) Positive urine microscopy. (3/19) Positive animal inoculation	65.2% (30/46)	-	19.6% (9/46)	(36/64) Cases excluded with no laboratory diagnosis
Van Riel J. Ann Soc Belg Med Tr. 1939 [47] (French)	Democratic Republic of the Congo 1937–8	Retrospective case series	32 (32/32) *	-	100% (32/32)	25 (mean) (10–63) (32/32)	(32/32) Single high titre MAT (Titre Not Specified)	65.6% (21/32)	-	9.4% (3/32)	No patients excluded
Vervoort H. Geneesk Tijdschr Ned-indië. 1923 [48] [Dutch]	Sumatra, Indonesia 1923	Prospective case Series	90 (90/90) *	-	-	-	(90/90) Blood culture positive	8.9% (8/90)	-	2.2% (2/90)	No patients excluded
Walch-Sorgdrager B. B World Health Organ. 1939 [49]	Netherlands 1924–39	Retrospective case series	12 (12/12) Canicola	-	-	-	(4/12) Positive urine culture. (12/12) Positive serology (>1:300)	0% (0/12)	-	0% (0/12)	No patients excluded
Wilmaers L & Renaux E. Arch Méd Belg. 1917 [50] [French]	Belgium 1916	Retrospective case series	22 (22/47) *	-	22/22 (100%)	- (7–13) (22/22)	(22/22) Isolation of leptospira	100% (22/22)	-	0% (0/22)	(25/47) Cases excluded as no laboratory diagnosis

When studies contained more than one patient series each series was displayed separately. All available data are included, but if data were not extractable it is indicated by a “-“. Percentage and (number/total number of patients) are quoted, but if no patient number was quoted then a “NA” is used. If no information on serovars is present a “*” is used.

### Study characteristics and assessment for bias

**Fig 2:**
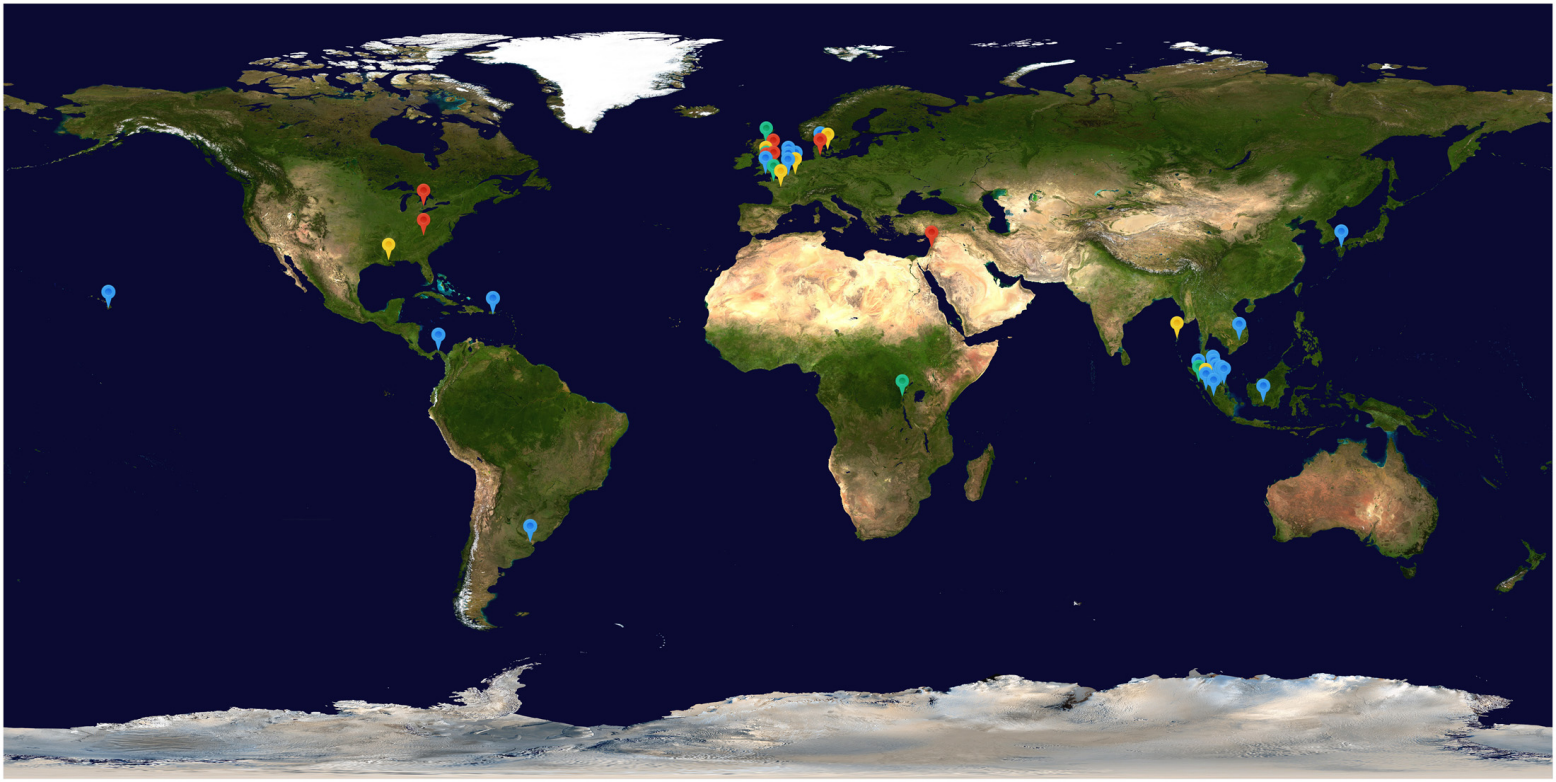
Location of patient series included in the review, colour coded according to series mortality. Blue = 0–5%, Green = 5–10%, Yellow = 10–20% and Red = >20%. Map image: NASA–Visible Earth.

Twenty-five articles were in English, 3 in Dutch, 3 in French, 2 in German, 1 in Spanish and 1 in Danish. The 41 patient series consisted of 25 retrospective patient series, 8 prospective patient series, 3 randomised control trials (RCTs), 3 non-randomised control trials (NRCTs) and 2 summaries of case reports, and were published between 1917 and 1984. Information on the location of the patient series was present for all case series (Fig 2). Eighteen series were located in Europe, 15 in Asia, 7 in the Americas and 1 in Africa. The median (range) number of patients in each series was 32 (10–459). All 41 patient series were designed to assess the clinical symptoms and outcome of leptospirosis. Each patient series was assessed for bias, and a summary of methodological quality across each criterion is displayed in S4 Fig, with further details for each series displayed in S5 and S6 Tables. Many patient series were limited by non-standardized study design and incomplete data. Diagnostic tests were at high risk of bias in 44% (18/41) of studies due to use of a single high admission titre with no confirmed cut-off titres, or no confirmed method of diagnosis for patients.

### Outcomes

**Fig 3:**
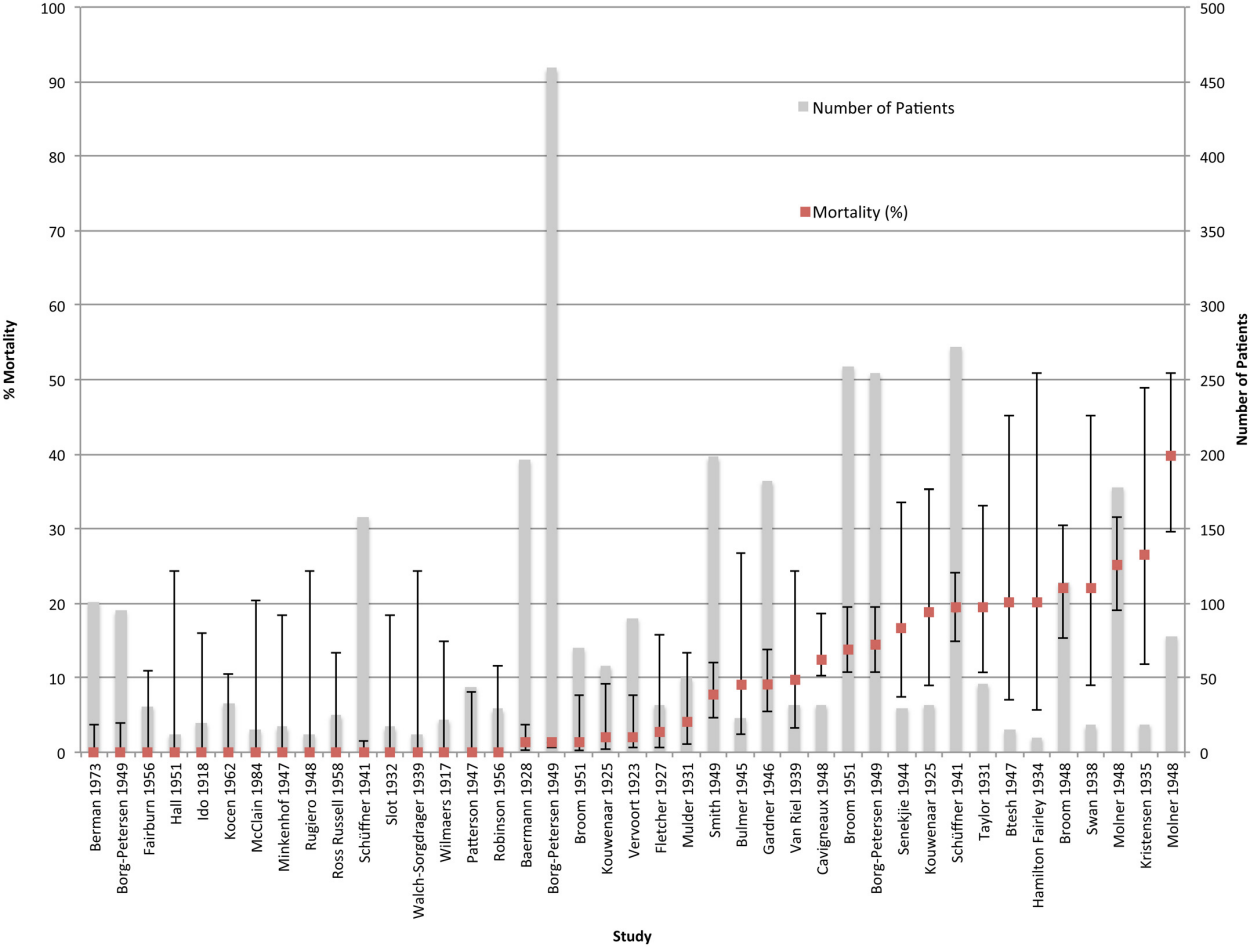
Mortality (%) (red line) and sample size (grey column) in order of increasing series mortality. 95% confidence intervals are estimated using the Wilson score interval [15].

Mortality data was available for all (41/41) patient series, for a total of 3,390 patients. Median series mortality was 2.2% (range 0–39.7%) with a total of 314/3,390 deaths, and a wide variation in mortality across series. No deaths were reported in 16/41 patient series, but a mortality of 20% or more was reported in 7/41 patient series (Fig 3). Data for demographics, secondary outcomes and laboratory results are displayed in Table 2, but were not available from all patient series. Information on secondary outcomes were described in between 2–38 (4.8–92.7%) studies for each outcome, but often had heterogeneous definitions or were not reported numerically, meaning that data could not be extracted from many articles. Available data showed that fever, headache and myalgia occurred in nearly all patients, while conjunctival suffusion was reported in over half of the patients. Haemorrhagic symptoms ranged from epistaxis to more severe bleeds, but details of haemorrhage were not always specified so it is not possible to report on the incidence of severe haemorrhage or SPHS.

**Table 2:** Demographics, clinical symptoms, and laboratory data in included patient series.

Criteria	Number of Patient Series (Of 41)	Number/ Total Number of Patients	Mean Series Value	Median Value Across Patient Series (Range)
Age	13	(860/3317)	32.3 years	36.7 years (15.3–41.3 years)
Sex	27	(2140/2571)	90.8% male	96.7% male (52.8–100% male)
Duration of Fever	11	(336/3317)	11.5 days	8.8 days (7.4–25.0 days)
Raised Temperature	21	(1312/1316)	99.9%	100% (96.0–100%)
Headache	13	(411/463)	83.6%	98.0% (25.0–100%)
Myalgia	17	(555/691)	80.4%	90.6% (12.0–100%)
Conjunctival Suffusion	17	(454/795)	59.0%	57.4% (23.5–100%)
Gastrointestinal symptoms	9	(117/228)	39.2%	33.0% (9.4–70.6%)
Haemorrhagic symptoms	16	(126/536)	26.0%	19.0% (2.0–70.0%)
Meningitis	18	(204/1130)	22.9%	12.2% (0.0–77.8%)
Jaundice	37	(1485/2957)	45.5%	22.8% (0.0–100%)
Hepatomegaly	7	(98/500)	28.0%	15.0% (6.7–90.0%)
Splenomegaly	8	(146/529)	16.8%	14.7% (0.0–48.0%)
Urea “Raised” *	12	(285/502)	61.3%	59.0% (0.0–100%)
WCC >10x10^9^/L	7	(115/301)	43.1%	43.9% (23.8–61.1%)
Bilirubin “Raised” **	2	(5/113)	2.3%	2.3% (0.0–4.6)

*Definition of raised urea ranged from urea >8.9mmol/L to >17mmol/L

**Definition of raised total bilirubin ranged from >25μmol/L to >85.5μmol/L

Mortality varied according to year and location of patient series and study design, with information present for all patient series (41/41). There was a wide range in mortality by year of study but no evident trend across time (S5 Fig). Between locations there was a wide range in mortality with median series mortality in Africa 9% (range n/a), 0.0% (range 0–39.7%) in the Americas, 1.0% (range 0–20%) in Asia, and 8.8% (range 0–26.3%) in Europe (S6 Fig). Median series mortality was high in 25 retrospective case series (8.0% (0.0–39.7)), and 2 patient series summarizing case reports (22.4% (20.0–24.8)) but lower in 6 controlled trials (0.0% (0–8.7%)) and 8 prospective case series (2.7% (0.0–19.6)) (S7 Table).

Median series mortality varied according to diagnostic grade and serovars. Mortality was lowest in patients with a grade I diagnosis and highest for those with a grade III diagnosis (Table 3). Across series with a grade II and III diagnosis the majority of patients were male and had a similar median age, while jaundice was highest in those with a grade III diagnosis, and lowest in those with a grade I diagnosis. Median series mortality was highest for serovar Icterohaemorrhagiae at 13.6% (0–34.3%), compared to 0.0% (0–50.0%) for Canicola and other serovars. Infections with serovar Icterohaemorrhagiae had a higher frequency of jaundice compared to other serovars (Table 4).

**Table 3:** Median series mortality stratified according to diagnostic grade.

	Number of Patient Series (Range—year of study)	Deaths / Number of Patients	Median Series Mortality (%) (Range %) [No. Patient Series]	Median Study % Jaundiced (Range %) [No. Patient Series]	Median Study % Male (Range %) [No. Patient Series]	Median Patient Age (years) (Range) [No. Patient Series]
Grade I	**11** (1917–1984)	21/554	**1.0** (0.0–20.0) [11]	**8.9** (0.0–100.0) [11]	**100** (100–100) [4]	**21.0** (21.0–21.0) [1]
Grade II	**11** (1931–1973)	82/660	**4.0** (0.0–39.7) [11]	**47.1** (0.0–100) [9]	**95.2** (93.6–100) [6]	**39.2** (34.4–41.25) [3]
Grade III	**19** (1934–1965)	211/2,176	**8.7** (0.0–26.3) [19]	**64.7** (0.0–100) [18]	**96.6** (52.8–100) [15]	**36.7** (15.3–38.7) [9]

**Table 4:** Mortality according to serovar.

Serovar	Number of Series	Median Series Mortality (%) (Range %)	Deaths / Number of Patients	Median % Jaundiced (Range %) [No. Patient Series]	% Male Median (Range %) [No. Patient Series]	Median Age (Range-Years) [No. Patient Series]
Ictero-haemorrhagiae	13	**13.6** (0.0–34.3) [13]	213/1,574	**84.4** (0–100) [12]	**96.6** (52.8–100) [10]	**34.4** (21.0–39.2) [7]
Canicola	7	**0.0** (0.0–50.0) [7]	4/204	**6.3** (0.0–100) [6]	**95.9** (93.6–100) [3]	**36.7** (21.0–39.2) [4]
Other serovars	8	**0.0** (0.0–20.0) [8]	9/582	**9.3** (0.0–100) [6]	**100** (100–100) [2]	**31.1** (21.0–41.3) [2]

### Patient characteristics

Data on mortality by age was available in 13/41 studies for 838 patients. Median series mortality was 0% (0–25%) in 7 series containing 51 patients aged 0–15, 16.3% (0–34.1%) in 11 series containing 308 patients aged 16–45, 36.7% (16.7–66.7%) in 6 series containing 70 patients aged 45–59, and 60.0% (33.3–60) in 3 series containing 23 patients aged over 60. Two large patient series, which were not incorporated due to differences in age stratification also showed a low mortality in children and a high mortality in older age groups. Smith [44] demonstrated a low mortality of 1% in 105 children aged 0–20 years compared to 50% in 18 patients aged 51 years or over, while Walch-Sorgdrager, [49] using the same cohort as Schuffner [41], demonstrated a mortality of 60% in 15 patients aged 60 years and over compared to 7.1% in 210 patients aged 10–40 years. Data on mortality by sex of patient was available in 21/41 patient series. Across 8 patient series the median series mortality for 227 female patients was 0% (range 0–40%), compared to 8.7% (range 0.0%- 39.7%) in 21 series containing 1077 male patients. No data was available on the untreated mortality of leptospirosis in pregnant women.

### Clinical symptoms

**Fig 4:**
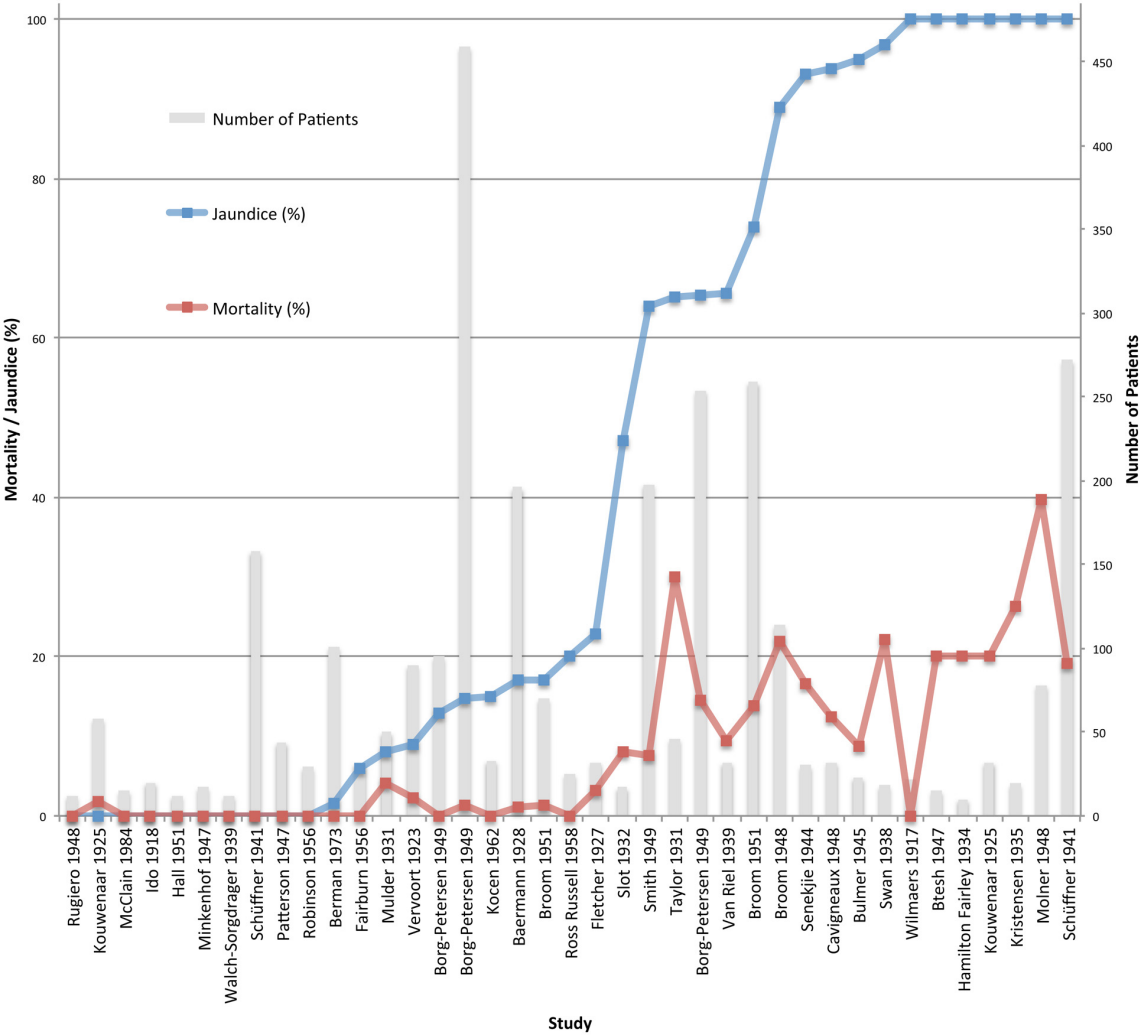
Mortality (red line) and number of patients (grey bar) by increasing frequency of jaundice (blue line).

Frequency of jaundice was reported in 37/41 patient series, ranged from 0% to 100%, and was associated with increased mortality (Fig 4). In 8 patient series where the incidence of jaundice was 0%, median series mortality was 0% (range 0–1.7%) with 0.3% (1/348) overall mortality; while in 9 patient series where incidence of jaundice was 100%, the median series mortality was 19.1% (range 0.0–39.7%), with 21.6% (143/662) overall mortality. Data on renal function was reported in 12/41 studies and mortality increased with higher frequency of renal failure. In 4 patient series, with a total of 137 patients, where 29.8% (0.0–44.9%) patients had renal failure, median series mortality was 0% (0–3.4%), while in 8 patient series, with a total of 349 patients, where 80.5% (52.0–100%) patients had renal failure; the median series mortality was 12.1% (range 0–25.0%). Data on mortality in patients with meningitis was present in 12/41 studies for 188 patients with 4 deaths reported overall. Median series mortality in patients with meningitis was 0% (range 0–25%), with a low median incidence of jaundice in these patients of 12.2% (0–100%).

## Discussion

This systematic review is the first to comprehensively evaluate available literature to define the untreated mortality from leptospirosis. The median mortality of leptospirosis across all patient series was 2.2%. The mortality range, however, was broad (0–39.7%), reflecting the wide clinical spectrum of disease severity alongside the heterogeneity of study designs, inclusion criteria, and diagnostic methods used. Median series mortality was lower than previous reports, which often cite series with a high mortality, such as those summarizing case reports. This is highlighted by the high median series mortality of 22.4% across series summarizing case reports in this review. Compiled results showed that leptospirosis normally causes uncomplicated, anicteric disease with around 10 days of fever and a low mortality of less than 1%. Uncomplicated disease is therefore a major cause of morbidity, an important contributor to DALYs, and a significant burden to local health resources, but is not usually fatal. In a minority of cases however, especially the older population, more severe disease complicated by jaundice, renal failure, meningitis and death can occur.

Mortality was associated with host factors; the median series mortality in series including jaundiced patients approached 20% and in those with a high incidence of renal failure was over 12%, although data on renal failure was less reliable. These findings are consistent with previous findings [4,6,51,52], that show severe complications like jaundice and renal failure are associated with a significantly higher mortality. Interestingly, the median series mortality in patients with meningitis was found to be low at 0%, with 2.1% (4/188) overall mortality. This contrasts with a recent study which suggested that meningitis is associated with a significant mortality [53].

Mortality increased with age and was highest in those aged over 60 (60.0% (33.3–60)) but was negligible in children under 15 years of age at 0% (0–25%), although patient numbers were small in these age groups. Previous studies have reported a low mortality in untreated children and an increased mortality in untreated patients over 40 years, and it is likely that co-morbidities that occur with age such as diabetes and renal failure, alongside immunosenescence, increase mortality, although these factors were not accounted for in these patients [8,54–56]. Recent research is not suggestive of significant differences in mortality between sexes and the fact that the majority of patients were male makes it hard to show any reliable difference in untreated mortality between sexes [8,56]. The predominance of male patients may be explained by occupational risk factors such as farming and military work, which are more common in the male population. No information on mortality in pregnant women was available in this review, but the relative immunosuppression of pregnancy is thought to increase complications and mortality from leptospirosis [57].

Mortality is thought to be affected by pathogen factors and in this study mortality varied according to infective serovar and location, although there was a wide variation within each continent and region. Mortality was higher in Europe and North America than in Asia, which may reflect the predominance of serovar Icterohaemorrhagiae in studies from these regions, as serovar Icterohaemorrhagiae had a higher mortality of 13.1% (range 0.0–34.3) and an increased frequency of jaundice 62.9% (range 0–100) compared to other serovars. These facts underline the importance of understanding the local epidemiology of disease to predict disease outcomes, although identification of serovar is often inaccurate, with one recent study showing that MAT only correctly identified serovar in 33% of cases [58]. Furthermore, current opinion is that specific serovars are not associated with particular disease types [6]. It is also possible that mortality is affected by the endemicity of leptospirosis, as a previous study has shown that morbidity and mortality are lower in pre-exposed populations compared to populations with no previous exposure [59].

There are several limitations to this study. Study selection bias is likely due to the limitations of electronic searches in old literature, the use of reference lists to identify articles, the exclusion of unobtainable studies, and the under representation of modern studies. Furthermore, some studies were excluded because data on treatment or diagnosis was incomplete, meaning that results from this study may not be representative of outcomes in all untreated patients. The use of only one author and of “Google Translate” for data extraction may have introduced inaccuracies, although all extracted data was checked for errors. Data extraction and analysis were hindered by the age of studies with baseline patient characteristics often absent, outcomes imprecisely reported or missing, and laboratory tests not performed, while extraction of untreated patients from a larger cohort may have led to further inaccuracies. Analysis was performed by patient series, rather than by study, which may have overestimated findings from studies containing more than one patient series.

The characteristics of study populations are important when interpreting results. This review excluded patients with asymptomatic disease and those with mild self-limiting disease who did not seek medical attention. It has been shown that a large proportion of people in endemic communities are seropositive for leptospirosis [59] but have not sought medical attention, meaning that many mild cases go undiagnosed and unrecorded. The lack of access to healthcare and diagnostics, especially in remote and resource-limited settings may also prevent patients with severe disease from obtaining medical care and lead to an underrepresentation of severe cases. Both of these factors are likely to influence the final estimate of the mortality from untreated disease. The increased life expectancy of modern populations and the fact that the large majority of patients included in this review were male (83.2% (2140/2571)), must also be taken into account when applying results to a wider context. Retrospective case series were more likely to include patients with severe disease and thus overestimate mortality, while 5 controlled trials published after 1950 excluded patients with severe disease and reported a mortality of 0%, which is likely to reflect their patient selection criteria, as it would have been unethical to include untreated patients with severe disease after the introduction of antibiotics in the 1940s. Diagnostic methods mean that included patients may not represent the overall population. Culture methods are likely to have a high specificity but low sensitivity and thus miss many diagnoses of leptospirosis, while serological tests have been shown to lack both sensitivity and specificity and therefore may not accurately diagnose leptospirosis [60].

### Conclusions and recommendations

Leptospirosis remains a major, under appreciated and under recognized infection whose burden of disease falls disproportionately on those in poor and developing regions of the world [61]. This review clarifies the untreated mortality from leptospirosis and shows that despite wide variation, it is of high significance in elderly, jaundiced patients and/or those with renal failure, but much lower in younger, anicteric patients. The results from this study will support the quantification of DALYs from leptospirosis and may be used to guide empirical treatment strategies. A greater understanding of the true incidence of disease through increased surveillance should be encouraged to understand the pathogenicity of local serovars and guide local empirical treatment strategies, while improved genotypic based tests are required to more accurately predict the virulence of local strains [62]. The development of accurate and inexpensive point-of-care antigen based diagnostic tests for the diagnosis of leptospirosis early in its disease course would prevent the development of complications or death in this easily treatable disease [60,63]. Strategies for managing the disease should stress the importance of early empirical treatment of fever with effective antibiotics such as penicillin and doxycycline, which are cheap and widely available.

## Supporting Information

S1 Checklist PRISMA (Preferred Reporting Items for Systematic Reviews and Meta-Analyses) checklist. (DOC)

S1 Fig Search results for Ovid MEDLINE database, 28^th^ July 2014. (TIF)

S2 Fig Search results for Embase Classic + Embase database, 28^th^ July 2014. (TIF)

S3 Fig Search results for Global Health database, 28^th^ July 2014. (TIF)

S4 Fig Percentage of patient series (41/41) within each criterion with high, medium and low bias. Green reports low bias, yellow medium bias and red high bias.
(TIF)

S5 Fig Untreated mortality (%) according to year of patient series. (TIF)

S6 Fig Median mortality according to location, with number of patient series in each country in brackets. Patient series are colour coded according to continent: Africa = dark blue, Americas = red, Asia = green and Europe = purple. Error bars show range when more than one patient series was performed in a country or continent
(TIF)

S1 Table Inclusion and exclusion criteria. (DOCX)

S2 Table Criteria for assessing bias within studies. (DOCX)

S3 Table Articles excluded due to the full text not being available or the article not being translated. (DOCX)

S4 Table Reasons for exclusion of full articles. (DOCX)

S5 Table Bias within each patient series. (DOCX)

S6 Table Assessment of bias within studies. (DOCX)

S7 Table Median mortality in patient series according to study design. (DOCX)
