# Supplementary figures and images for: A Systematic Review of the Mortality from Untreated Leptospirosis
Source: PLoS Negl Trop Dis. 2015 Jun 25;9(6):e0003866. doi: 10.1371/journal.pntd.0003866 (PMC4482028; doi:10.1371/journal.pntd.0003866)

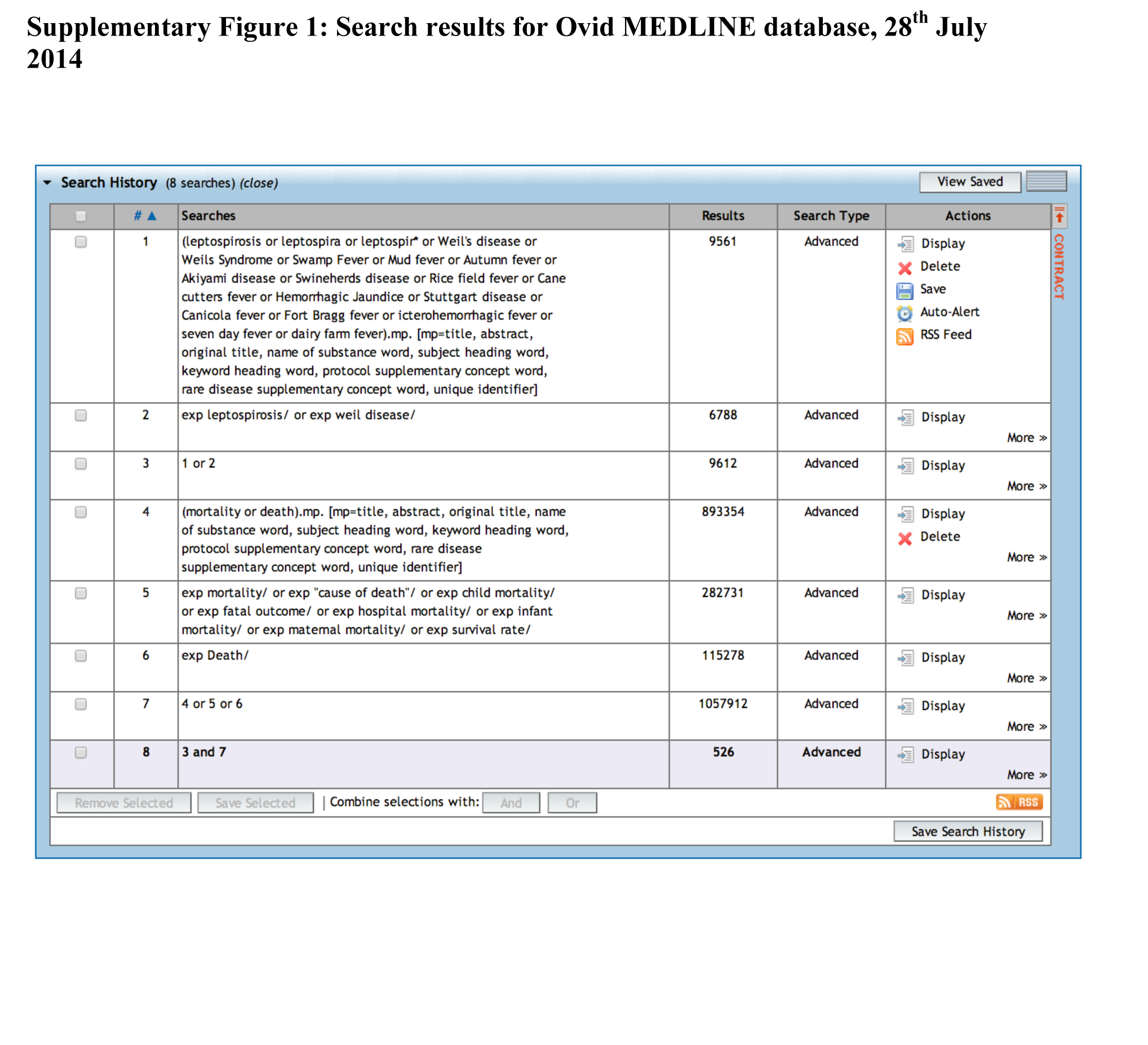

Supplement: S1 Fig — (TIF) [file pntd.0003866.s002.tif]

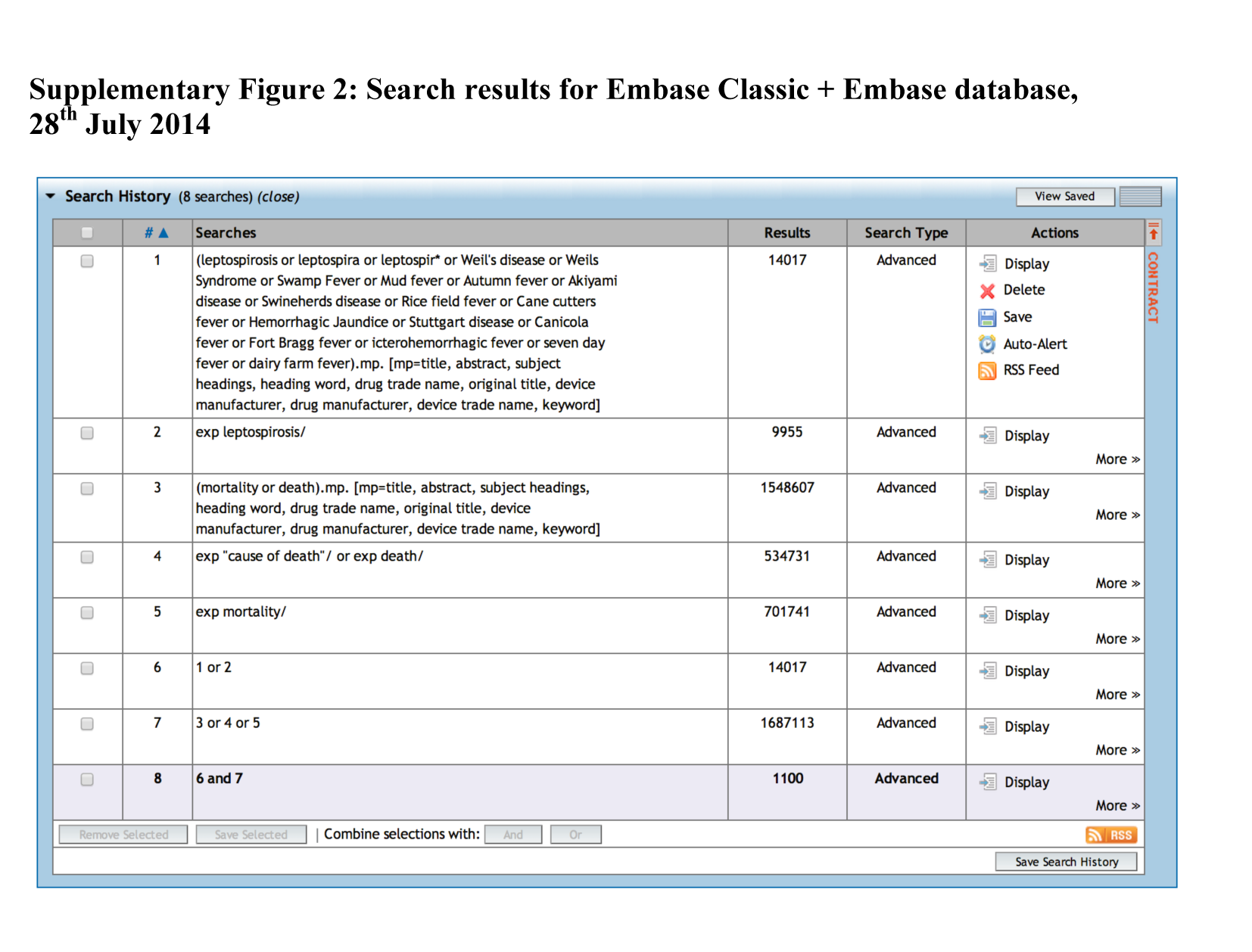

Supplement: S2 Fig — (TIF) [file pntd.0003866.s003.tif]

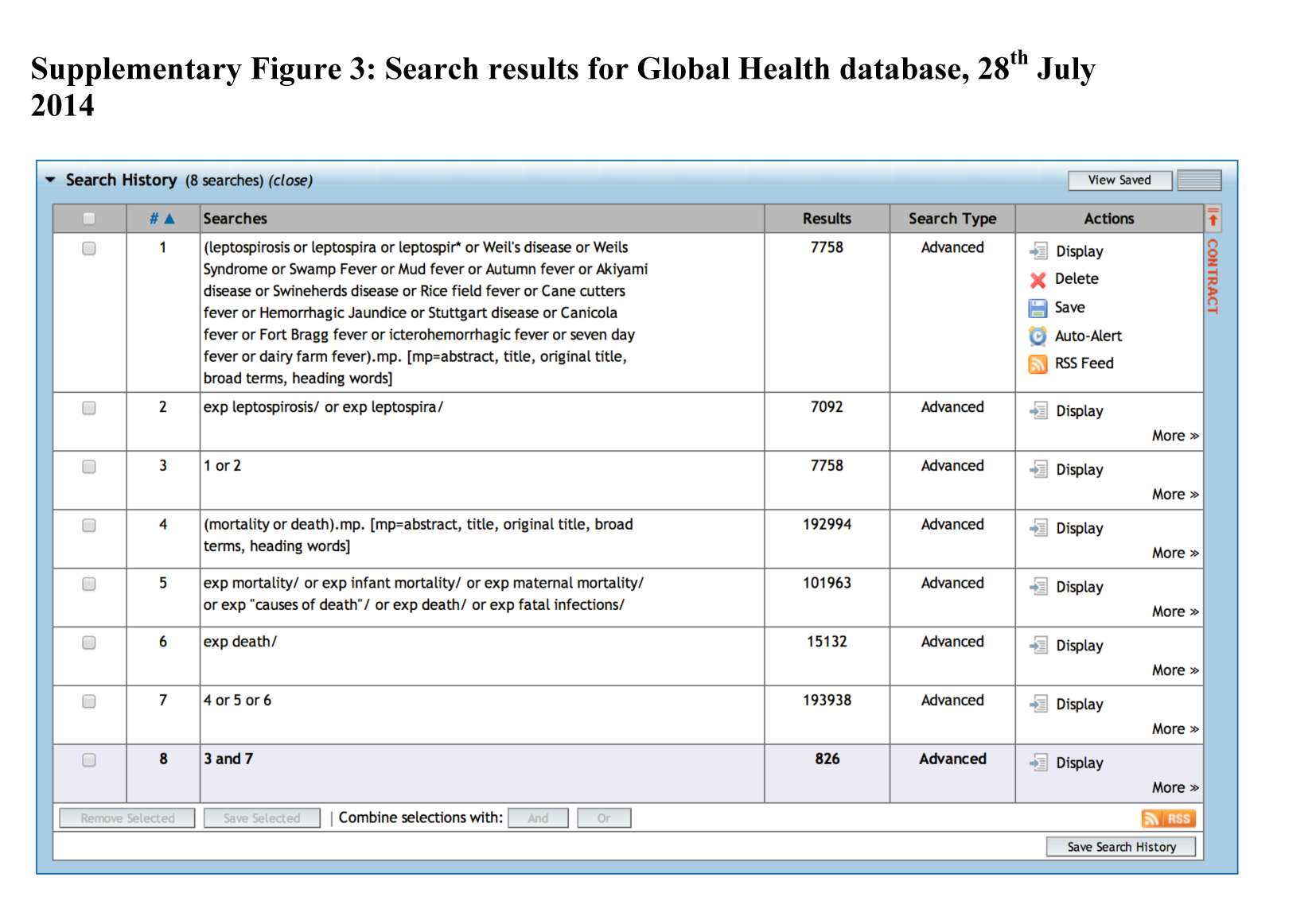

Supplement: S3 Fig — (TIF) [file pntd.0003866.s004.tif]

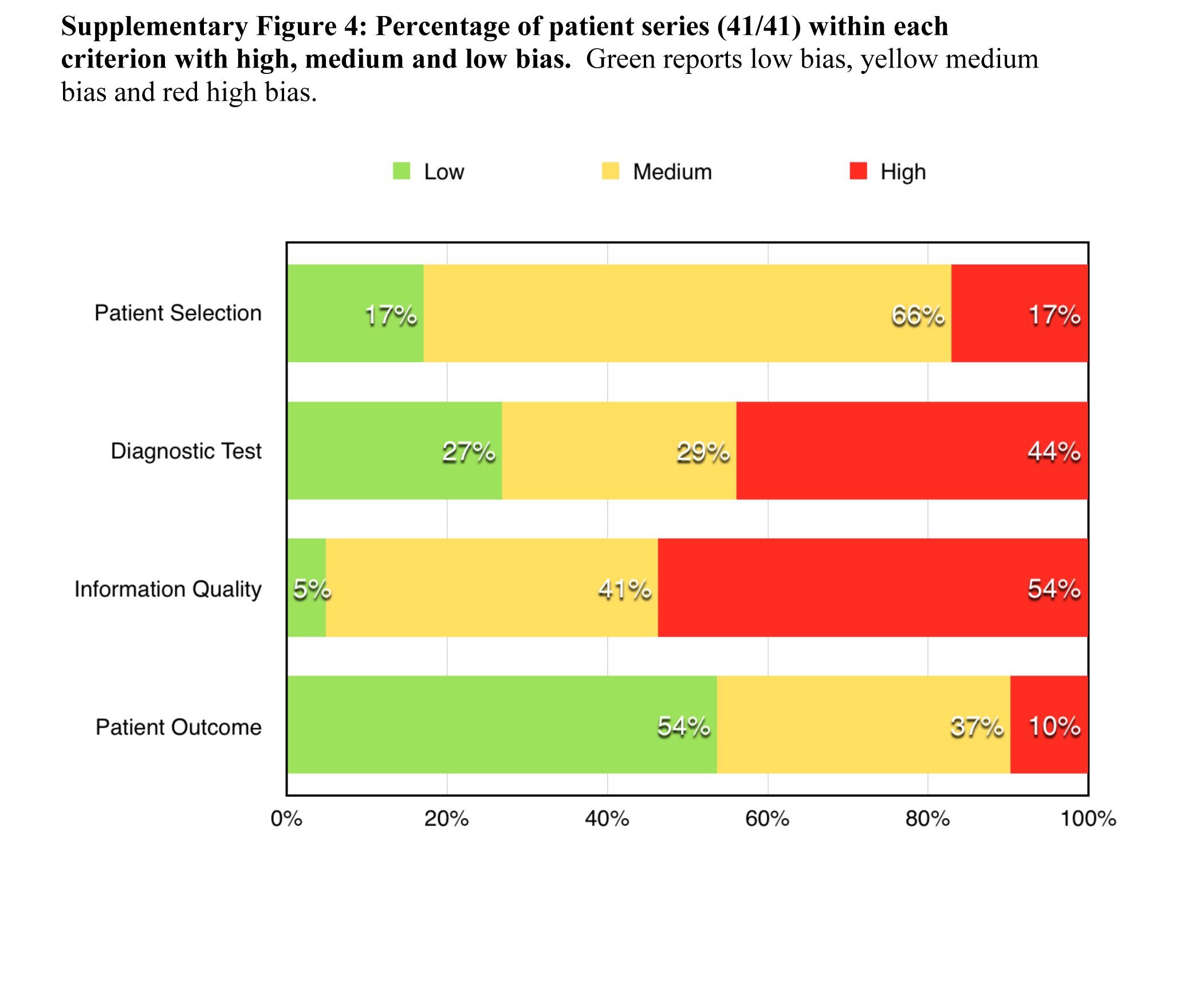

Supplement: S4 Fig — Green reports low bias, yellow medium bias and red high bias. (TIF) [file pntd.0003866.s005.tif]

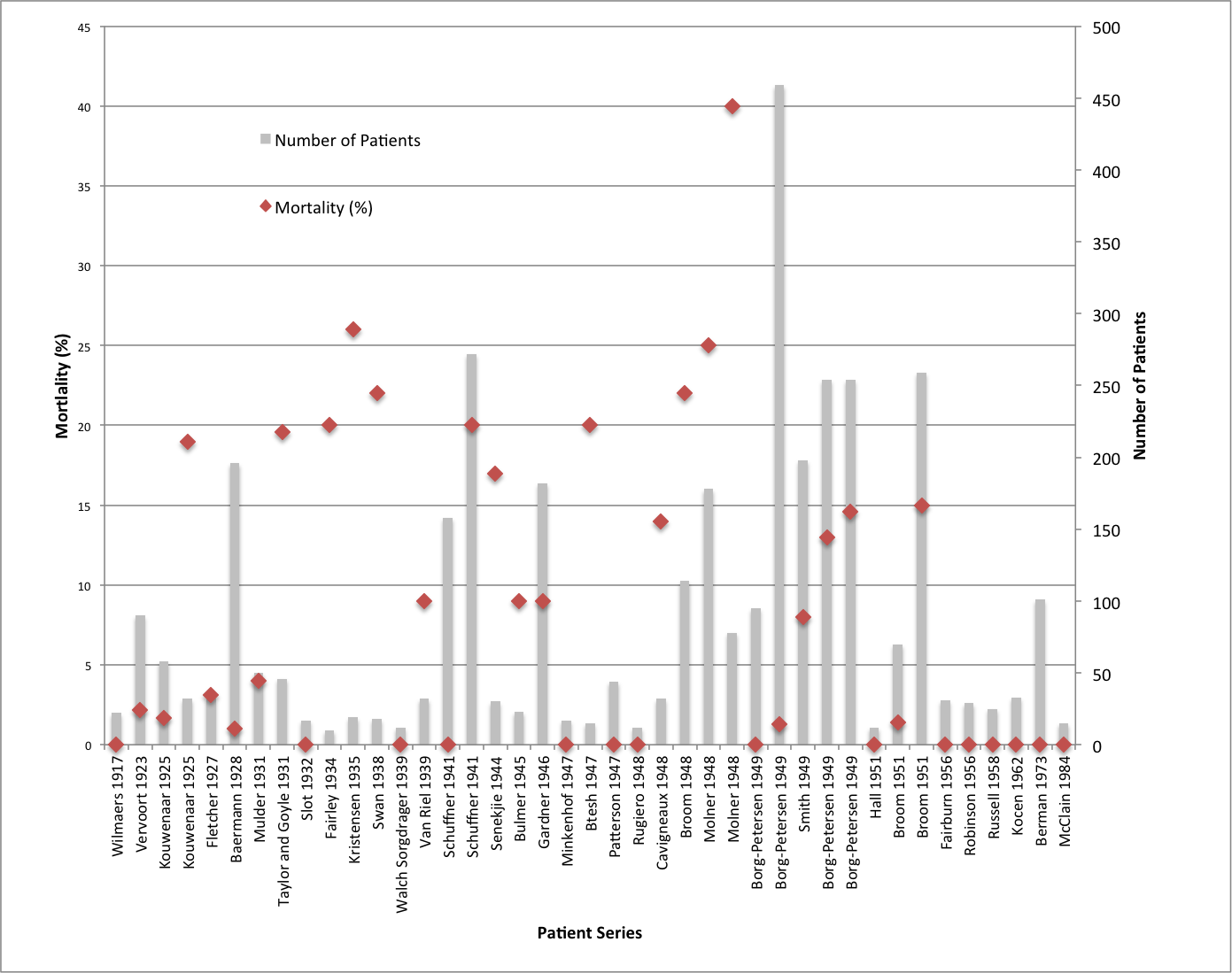

Supplement: S5 Fig — (TIF) [file pntd.0003866.s006.tif]

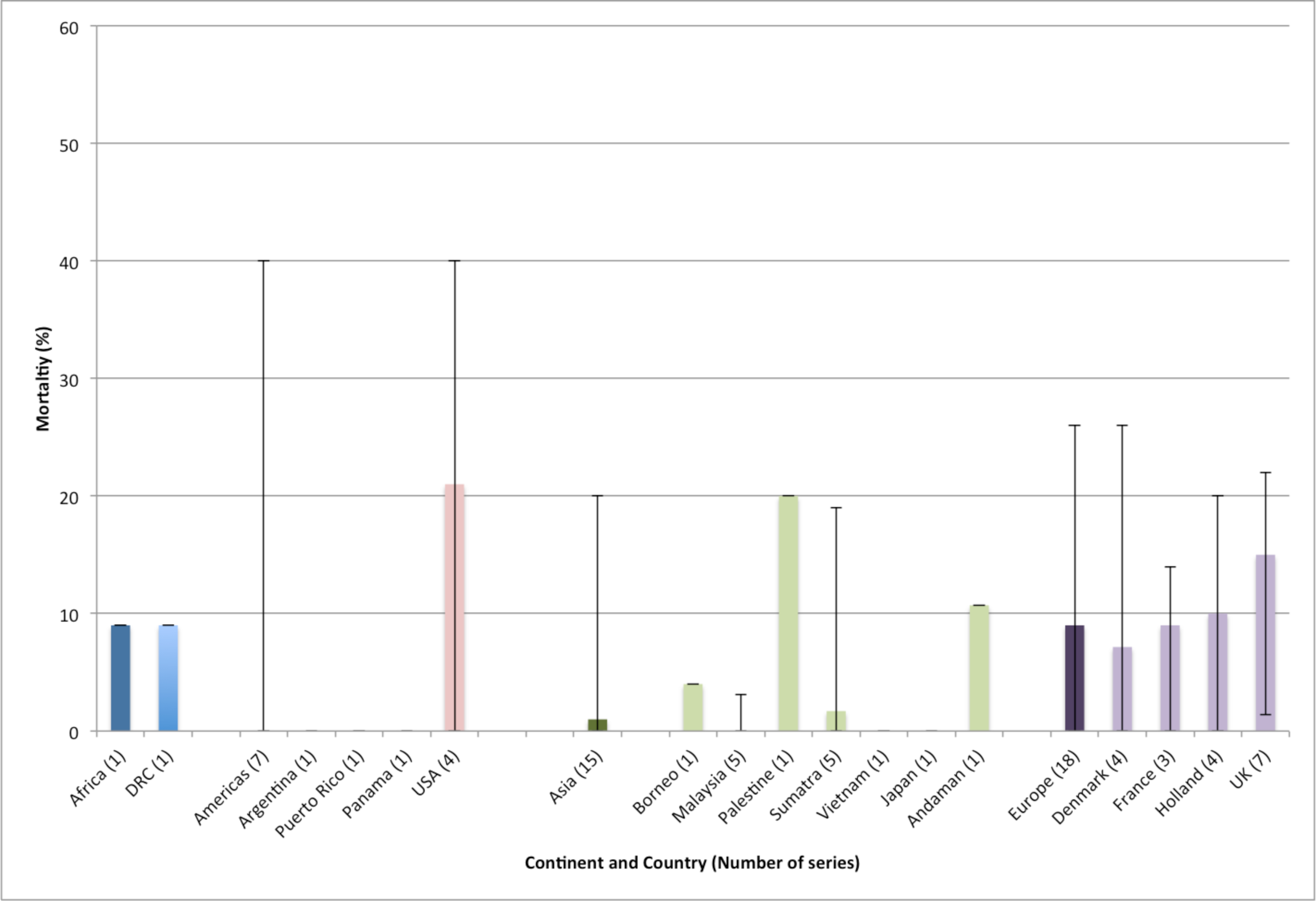

Supplement: S6 Fig — Patient series are colour coded according to continent: Africa = dark blue, Americas = red, Asia = green and Europe = purple. Error bars show range when more than one patient series was performed in a country or continent (TIF) [file pntd.0003866.s007.tif]
