# Supplementary material for: A Systematic Review of the Mortality from Untreated Leptospirosis
Source: PLoS Negl Trop Dis. 2015 Jun 25;9(6):e0003866. doi: 10.1371/journal.pntd.0003866 (PMC4482028; doi:10.1371/journal.pntd.0003866)
Supplement: S1 Table — (DOCX) [file pntd.0003866.s008.docx]

### Supplementary Table 1: Inclusion and exclusion criteria

|  | **Inclusion Criteria** | **Exclusion Criteria** |
| --- | --- | --- |
| **Participants** | - All ages and both sexes - Symptomatic patients | - Asymptomatic patients |
| **Interventions** | - IV fluids - Hospital care | - Antibiotic treatment - ICU treatment - Dialysis - Serum therapy |
| **Diagnosis** | - Culture - Microscopy - PCR - MAT | - Clinical diagnosis only |
| **Study Design** | - All study designs - All locations - All languages - All years of publication | - Less than 10 patients in series |
