# Supplementary material for: A Systematic Review of the Mortality from Untreated Leptospirosis
Source: PLoS Negl Trop Dis. 2015 Jun 25;9(6):e0003866. doi: 10.1371/journal.pntd.0003866 (PMC4482028; doi:10.1371/journal.pntd.0003866)
Supplement: S2 Table — (DOCX) [file pntd.0003866.s009.docx]

**Supplementary Table 2: Criteria for assessing bias within studies**

| **Category** | **Grade** | **Criteria** |
| --- | --- | --- |
| **Patient Selection** | Grade I | -Prospective, consecutive patient case series with no inappropriate exclusions. |
|  | Grade II | -Prospective, non-consecutive case series  -Retrospective case series  -Reference laboratory series |
|  | Grade III | -Exclusion of patients (i.e. most unwell patients treated) likely to significantly affect outcome  -Summary of case reports from literature. |
|  |  |  |
| **Diagnostic Test** | Grade I | -Direct observation of leptospires in blood or urine, or by culture of leptospires though culture medium or animal inoculation.  -Serological diagnosis with either sero-conversion or fourfold antibody response |
|  | Grade II | -Single high MAT titre of ≥1:400 in an endemic region, or ≥1:100 in a non-endemic region |
|  | Grade III | -Single high MAT titre but titre non-significant for all patients  -No record of significant MAT titre  -Diagnosis confirmed but no record of diagnostic technique or titre for serological tests. |
|  |  |  |
| **Missing information** | Grade I | -No information missing |
|  | Grade II | -Information missing on **1 or 2** of Age / Sex / Renal Status / Jaundice / Mortality |
|  | Grade III | -Information missing on **3** or more of Age / Sex / Renal Status / Jaundice / Mortality |
|  |  |  |
| **Outcome** | Grade I | -Known for all patients. No patients excluded |
|  | Grade II | -Outcome not known for all patients  -Patients excluded |
|  | Grade III | -Outcome not included for all patients in series due to treatment |
