# Supplementary material for: A Systematic Review of the Mortality from Untreated Leptospirosis
Source: PLoS Negl Trop Dis. 2015 Jun 25;9(6):e0003866. doi: 10.1371/journal.pntd.0003866 (PMC4482028; doi:10.1371/journal.pntd.0003866)
Supplement: S3 Table — (DOCX) [file pntd.0003866.s010.docx]

### Supplementary Table 3: Articles excluded due to the full text not being available or the article not being in English (n=9) – see also Figure 1 (flowchart)

| **Paper** | **Reason not included** |
| --- | --- |
| Bals M, Serbanescu FL, P S, Tofan N, Caruntu F, Roman A, et al. The clinical picture of leptospirosis in the region of Bucharest. III. Aspectul clinic al leptospirozelor din regiunea Bucuresti. III. Probl Tuberk. 1958;9(2):131–6. | Paper not obtained |
| Blagoveschenskaya NM. Epidemiology of leptospirosis without jaundice. Z Mikrobiol. 1957;2:82–7. | Paper not translated |
| Chumakov ME. Leptospirosis in the Republic of Mordovia. Med Parazitol (Mosk). 2004;4:45–50. | Paper not translated |
| Danaraj TJ. Leptospirosis. Proc Alumni Assoc King Edward VII Coll Med Singapore. 1950;3:326. | Paper not obtained |
| Hugonot, Delons, Moulay-Idriss. Etudes sur les leptospiroses. Le rein des leptospirosies. La leptospirose des hammams. Sem des Hop. 1962;82. | Paper not obtained |
| Lemierre A. Maladies infectieuses. Masson edit, Paris. 1937. | Paper not obtained |
| Popov VA, Efremenko VI, Antonenko AD, Nadeina VP, Kovalev NG, Grizhebovskii GM, et al. Epidemiological features of leptospirosis in Stavropol’ region. Zhurnal Mikrobiol Epidemiol i Immunobiol. 2001;6 suppleme:74–6. | Paper not translated |
| Schmitt N, Warburg IW. Swamp fever among farmers. Mice as carriers of leptospirosis. (Landleute erkranken an feldfieber-maus als ubertrager der leptospire. ). Schadlingsbekampfung, Staufen Im Br. 1951;43(2):42–3. | Paper not obtained |
| Zeevi A. On an Epidemi of Leptospirosis in a rural Area. Dapim Reffuim. 1950;9(2):iii. | Paper not obtained |
