# Supplementary material for: A Systematic Review of the Mortality from Untreated Leptospirosis
Source: PLoS Negl Trop Dis. 2015 Jun 25;9(6):e0003866. doi: 10.1371/journal.pntd.0003866 (PMC4482028; doi:10.1371/journal.pntd.0003866)
Supplement: S4 Table — (DOCX) [file pntd.0003866.s011.docx]

### Supplementary Table 4: Reasons for exclusion of full articles

| **Study Title** | **Inclu-ded** | **Excluded** | | | | | | | **Notes** |  |  |
| --- | --- | --- | --- | --- | --- | --- | --- | --- | --- | --- | --- |
|  |  | **No Laboratory diagnosis** | **Treated Abx / Serum / No information on treatment** | **<10 Patients** | **Not enough Information on Treatment, Diagnosis, Outcome** | **Duplicated Patient Series** | **On ICU or Received Dialysis** | **Asymptomatic** |  |  |  |
| Agampodi, S.B. et al., 2011. Leptospirosis outbreak in Sri Lanka in 2008: lessons for assessing the global burden of disease. American Journal of Tropical Medicine and Hygiene, 85(3), pp.471–478. | N | N | Y | N | N | N | N | N | "Patients treated as leptospirosis by physicians" - no record of which antibiotic |  |  |
| Alicata & J. E., 1949. Observations on Leptospiral Infections in Samoa. Hawaii Medical Journal, Nov.-Dec. (2), pp.85–9. | N | N | N | Y | Y | N | N | N | No case series. "No human cases yet proven in Samoa" |  |  |
| Alston, J.M., 1935. Leptospiral Jaundice among Sewer-Workers. Lancet, April 6. (5823), pp.806–9. | N | N | N | Y | N | N | N | N | Only 3 cases (1 death) |  |  |
| Anusz, Z., 1968. Sytuacia Epidemiologiczna Leptospiroz w swiecie ze szczegolnym uwzglednieniem Polski w Latach. Przeglad Epidemiologiczny, 22(1), pp.45–50. | N | N | ? | N | Y | N | N | N | No information on treatment or outcomes |  |  |
| Arias P, H. et al., 2003. Outbreak of leptospirosis in children in Linares. Revista Chilena de Pediatria, 74(4), pp.405–410. | N | N | Y | N | N | N | N | N | Treated with Amoxicillin |  |  |
| Ashe, WM., Pratt-Thomas, HR., Kumpe, C., 1941. Weil’s Disease. Medicine, 20, pp.145–210. | N | N | N | Y | Y | Y | N | N | Review describes cohorts previously described in other case series (Leptospiroses Walch-Sorgdrager B., Bull. Health Organization (League of Nations) 8:144-346. 1939.) Only 7 cases described in the author's cohort. |  |  |
| Ashford, D. a et al., 2000. Asymptomatic infection and risk factors for leptospirosis in Nicaragua. The American journal of tropical medicine and hygiene, 63(5-6), pp.249–54. | N | N | N | N | Y | N | N | Y | Mostly asymptomatic infections. Seroprevalence study |  |  |
| Austoni, M., 1952. Leptospirosis in the Padua district. Clinical and epidemiological notes on 280 cases. A report. Le leptospirosi nella zona padovana. Rilievi clinici ed epidemiologici su 280 casi (Relazione). Giornale di Malattie Infettive e Parassitarie, 4(6), pp.323–336. | N | N | ? | N | Y | N | N | N | No clear information on treatment. |  |  |
| Bacalbasa, B. & Vacs, L., 1969. Contributions to the clinical study of leptospirosis in the county of Galati. <Contributii la studiul clinic al leptospirozei in judetul Galati.>. Viata Medicala, 16(11), pp.739–746. | N | N | ? | N | Y | N | N | N | No clear information on treatment. (Romanian) |  |  |
| Baermann, G., 1923. Clinical and Experimental Investigations into Spirochaetal Diseases and spirochaetes at Deli. Geneeskundig Tijdschrift voor Nederlandsche-Indie, 63(6), pp.885–921. | N | N | N | Y | N | N | N | N | Excluded - only 8 patients |  |  |
| Bedernjak, J., 1994. Leptospirosis in Pomurje and Slovenia. Orvosi hetilap, 135(8), p.409. | N | N | ? | N | Y | N | N | N | No clear information on treatment (Hungarian). |  |  |
| Bezemer F. De ziekte van Weil op celebes. Geneeskd Tijdschr voor Ned. 1933;1194. | N | N | N | Y | N | N | N | N | 5 cases only. |  |  |
| Bharadwaj, R. et al., 2002. An urban outbreak of leptospirosis in Mumbai, India. Japanese journal of infectious diseases, 55(6), pp.194–196. | N | Y | ? | N | Y | N | N | N | No clear treatment information. |  |  |
| Blanchard, M., Lefrou, G. & Laigret, J., 1934. Spirochétose ictérigène épidémique observée sur des indigènes à Brazaville. Bulletin de la Societe de pathologie exotique, 16(3), pp.184–193. | N | Y | N | N | Y | N | N | N | Spirochetes only demonstrated in 5/15 cases. |  |  |
| Brown, H., 1928. Epidemic Jaundice in the Andaman Islands. Lancet, 211(5452), p.388. | N | N | N | Y | N | N | N | N | Single case. Confirmation of existence of leptospirosis on the Andaman Islands. |  |  |
| Brown, M.G. et al., 2010. Leptospirosis in suspected cases of dengue in Jamaica, 2002-2007. Tropical Doctor, 40(2), pp.92–94. | N | N | ? | N | Y | N | N | N | No information on treatment or outcome |  |  |
| Brown, G.W. et al., 1984. Febrile Illness in Malaysia - An analysis of 1,629 hospitalized patients. Am. J. Trop. Med. Hyg., 33(2), pp.311–315. | N | N | ? | N | Y | N | ? | N | Patients' treatment not specified |  |  |
| Bruce, M.G. et al., 2005. Leptospirosis among patients presenting with dengue-like illness in Puerto Rico. Acta tropica, 96(1), pp.36–46. | N | N | ? | N | Y | N | N | N | "Detailed history of antibiotic use in hospitalized patients was not obtained |  |  |
| Bruno, F., Wilen, C. & Snavely, J., 1943. Spirochaetal Jaundice. A Report on Fifteen Cases, including Two Cases of Leptospira Canicola Infection. JAMA (Chicago, Ill.), 123(9), pp.519–24. | N | N | N | Y | N | N | N | N | Only 3 patients |  |  |
| Buzzard EM, Wylie JAH. Meningitis Leptospirosa. Lancet. 1947;417–20. | N | N | N | Y | N | N | N | N | Only 5 patients. All untreated and no deaths. |  |  |
| Caldas, E.M. et al., 1979. Epidemiologic study of the leptospirosis outbreak occurring in Salvador, Bahia, in May and June 1978. Revista do Instituto Adolfo Lutz, 39(1), p.85. | N | N | ? | N | Y | N | N | N | No clear information on treatment but likely to have been given antibiotics. |  |  |
| Carles, G. et al., 1995. Leptospirosis and pregnancy. A series of 11 cases in French Guiana. Journal de Gynecologie Obstetrique et Biologie de la Reproduction, 24(4), p.418. | N | N | Y | N | N | N | N | N | 10/11 patients treated with antibiotics. |  |  |
| Cecilia Perret, P. et al., 2005. Prevalencia y presencia de factores de riesgo de leptospirosis en una población de riesgo de la Región Metropolitana. Rev Méd Chile, 133, pp.426–431. | N | N | N | N | Y | N | N | Y | Serological survey |  |  |
| Cengiz, K. et al., 2002. Acute renal failure in leptospirosis in the black-sea region in Turkey. International urology and nephrology, 33(1), pp.133–6. | N | N | Y | N | N | N | Y | N | All patients treated with penicillin. 48% treated with haemodialysis |  |  |
| Ciceroni, L. et al., 2000. Epidemiological trend of human leptospirosis in Italy between 1994 and 1996. European journal of epidemiology, 16(1), pp.79–86. | N | N | ? | N | Y | N | N | N | No information on treatment |  |  |
| Clerke A.M., Leuva A.C., Joshi C., Trivedi S.V. 2002.Clinical profile of leptospirosis in South Gujarat.Journal of Postgraduate Medicine. 48 (2) (pp 117-118), | N | N | ? | N | Y | N | Y | N | No clear information on treatment but likely to have been given antibiotics (managed conservatively). 2 patients received haemodialysis. |  |  |
| Cochez P, Fichet. Nouvelles observations de Spirochétose méningé anictérique. Presse Med. 1933;32:647. | N | N | N | Y | N | N | N | N | 3 patients. All untreated. |  |  |
| Correa, M., 1969. Leptospiroses in Sao Paulo, Brazil. Revista do Instituto Adolfo Lutz, 29/30(1), pp.29–37. | N | N | ? | N | Y | N | N | N | No information on treatment or diagnosis. |  |  |
| Costa, E., Lopes, A.A. & Sacramento, E., 2001. Severe forms of leptospirosis: clinical, demographic and environmental aspects. Revista da Sociedade Brasileira de Medicina Tropical, 34(3), pp.261–267. | N | N | Y | N | N | N | Y | N | High proportion of patients treated with dialysis or Antibiotics. No separate untreated cohort |  |  |
| Costa, E. et al., 2003. Penicillin at the late stage of leptospirosis: a randomized controlled trial. Revista do Instituto de Medicina Tropical de São Paulo, 45(3), pp.141–5. | N | N | N | N | N | N | Y | N | 18% patients received dialysis in untreated cohort |  |  |
| Cotter, T.J., 1936. Weil’s Disease in North Queensland. BMJ, Supplement, pp.51–56. | N | Y | N | N | Y | N | N | N | No clear diagnosis. Initial outbreaks confirmed "clinically". Later outbreaks were able to isolate leptospires from some patients. |  |  |
| Covic, A. et al., 2003. A retrospective 5-year study in Moldova of acute renal failure due to leptospirosis: 58 cases and a review of the literature. Nephrology Dialysis Transplantation, 18(6), pp.1128–1134. | N | N | Y | N | N | N | Y | N | All patients on dialysis |  |  |
| Cumming, H.S., 1934. La spirochétose ictérohémorrhagic (maladie de Weil) et les ictères infectieux aux états-unis. Bulletin de l’Office International d'Hygiene Publique, 26, pp.1749–56. | N | Y | N | N | Y | N | N | N | No clear patient information and diagnosis. |  |  |
| Da Silva, J. et al., 1968. Estudo Prelimar das Leptospirosis no estado do Rio de Janeiro. Revista da Sociedade Brasileira de Medicina Tropical, 2(6), pp.317–37. | N | N | ? | N | Y | N | N | N | No information on treatment (Portuguese) |  |  |
| Daher, E, Trevisan Zanetta, D M, Cavalcante, M B, and Abdulkader, R.C.R.M., 2012. Risk Factors for death and changing patterns in leptospirosis acute renal failure. Am. J. Trop. Med. Hyg., 61(4), pp.630–634. | N | N | Y | N | N | Y | Y | N | High proportion of patients treated with dialysis or Antibiotics. No separate untreated cohort |  |  |
| Daher, E.F. et al., 2012. Leptospirosis-associated acute kidney injury: penicillin at the late stage is still controversial. Journal of clinical pharmacy and therapeutics, 37(4), pp.420–5. | N | N | N | N | N | N | Y | N | Patients received dialysis (49% of untreated cohort) |  |  |
| Daher, E.F. et al., 2009. Predictors of oliguric acute kidney injury in leptospirosis. A retrospective study on 196 consecutive patients. Nephron. Clinical practice, 112(1), pp.c25–30. | N | N | N | N | N | N | Y | N | Renal dialysis for many patients (52%) |  |  |
| Daher, E.F. et al., 2011. Different patterns in a cohort of patients with severe leptospirosis (Weil syndrome): effects of an educational program in an endemic area. The American journal of tropical medicine and hygiene, 85(3), pp.479–84 | N | N | N | N | Y | N | N | N | Renal dialysis for many patients (24-75%). Antibiotics for many patients. |  |  |
| Daher, E. et al., 2010. Clinical presentation of leptospirosis: A retrospective study of 201 patients in a metropolitan city of Brazil. Brazilian Journal of Infectious Diseases, 14(1), pp.3–10. | N | N | Y | N | N | N | Y | N | Majority of patients treated with dialysis and antibiotics |  |  |
| Daher, E.F., Nogueira, C.B., 2000. Evaluation of penicillin therapy in patients with leptospirosis and acute renal failure. Rev. Inst. Med. trop. S. Paulo, 42(6), pp.327–332. | N | N | N | N | N | N | Y (50%) | N | Significant proportion required dialysis |  |  |
| Dassanayake, D.L.B. et al., 2012. Predictors of the development of myocarditis or acute renal failure in patients with leptospirosis: An observational study. BMC Infectious Diseases, 12, p.4. | N | N | ? | N | Y | N | N | N | No information on treatment |  |  |
| Davidson, L.S.P. & Smith, J. 1939. Weil’s disease in the North-East of Scotland. BMJ, 2(4110), pp.753–757. | N | N | N | N | N | Y | N | N | Repeated Cohort “ Smith, J., 1949. Weil’s Disease in the North-East of Scotland. Brit. J. Indust. Med., 6(4), pp.213–220.” |  |  |
| Davidson. L.S.P., Campbell, R.M, Rae, H.J, Smith, J., 1934. Weil’s Disease (Leptospirosis). British Medical Journal, 2(3859), p.1138. | N | N | N | N | N | Y | N | N | Repeated Cohort “Smith, J., 1949. Weil’s Disease in the North-East of Scotland. Brit. J. Indust. Med., 6(4), pp.213–220.” |  |  |
| Davidson, L., 1938. Weil’s Disease: a New Occupational Disease in Fish Workers. Glasgow Medical Journal, 11, pp.113–122. | N | N | N | N | N | Y | N | N | Repeated Cohort “Smith, J., 1949. Weil’s Disease in the North-East of Scotland. Brit. J. Indust. Med., 6(4), pp.213–220.” |  |  |
| Dawson, B., Hume, W.E., Bedson, S.P., 1917. Infective jaundice. BMJ, 2(2959), pp.345–354. | N | Y | N | Y | Y | N | N | N | No clear diagnosis for patients. No clear cohort of patients |  |  |
| De Azevedo, R. & Correa, M., 1968. Leptospirosis: epidemiological clinical and laboratory studies of an outbreak observed in Recife, Pernambuco, Brazil, in 1966. Revista do Instituto Adolfo Lutz, 28(1), pp.85–111. | N | N | Y | N | N | N | N | N | Treated with tetracycline antibiotics |  |  |
| De Lajudie P, Brygoo E-R. Les Leptospiroses au Vietnam. Med Trop. 1953;13:466. | N | Y | N | N | Y | N | N | N | No clear diagnosis for all patients. No record of treatment given. |  |  |
| DebMandal, M., Mandal, S. & Pal, N.K., 2011. Serologic evidence of human leptospirosis in and around Kolkata, India: a clinico-epidemiological study. Asian Pacific journal of tropical medicine, 4(12), pp.1001–6. | N | N | Y | N | N | N | ? | N | All treated with antibiotics |  |  |
| Dechet, A.M. et al., 2012. Leptospirosis outbreak following severe flooding: a rapid assessment and mass prophylaxis campaign; Guyana, January-February 2005. PloS one, 7(7), p.e39672. | N | Y | Y | N | Y | N | N | N | Most patients treated with prophylactic doxycycline. |  |  |
| Derrick, E.H., 1957. Leptospirosis in North Queensland. Papua and New Guinea medical journal, 2(2), pp.23–24. | N | N | Y | N | N | N | N | N | Majority of patients treated with antibiotics. |  |  |
| Doherty, R.L., 1955. A Clinical Study of Leptospirosis in North Queensland. Australasian Ann. Med., 4(1), pp.53–63. | N | N | Y | Y | N | N | N | N | Only 8 untreated patients out of 112. |  |  |
| Drenowski, A.K., 1947. A Small Outbreak of Weil’s Disease in Bulgaria. Schweizerische Medizinische Wochenschrift, 77(51), p.1333. | N | Y | N | N | Y | N | N | N | No clear diagnosis. (German) |  |  |
| Drenovski, A., 1947. An epidemic of Weil’s disease in Bulgaria. (Une epidemie de morbus Weil en Bulgarie). Bruxelles medical., 27(17), p.957. | N | Y | N | N | Y | Y | N | N | No Clear Diagnosis for patients. (French) Repeat cohort of "Drenowski, A.K., 1947. A Small Outbreak of Weil’s Disease in Bulgaria. Schweizerische Medizinische Wochenschrift, 77(51), p.1333." |  |  |
| Drew, J.G., 1934. An account of Weil’s Disease in Queensland. BMJ, Dec 22. (3859), pp.1142–1143. | N | Y | N | N | Y | N | N | N | No clear diagnosis |  |  |
| Durich, J., 1953. Leptospirosis among the workers in the rice fields. La leptospirosis entre los cultivadores de los arrozales. Revista de sanidad e higiene publica, 27(3-4), pp.179–216. | N | N | Y | N | N | N | N | N | Majority of patients treated with antibiotics. No separate outcome information for untreated cohort. (Spanish) |  |  |
| Durich, J. & Pumarola, A., 1956. Leptospirosis in the province of Valencia. Results of the vaccination campaign in 1955. La leptospirosis en la provincia de Valencia. Resultados de la campana de vacunacion de 1955. Revista de sanidad e higiene publica, 30(3), pp.201–207. | N | N | Y | N | N | N | N | N | Majority of patients treated with antibiotics. No separate outcome information for untreated cohort. (Spanish) |  |  |
| Edwards, CN, Nicholson, GD, Hassell, T., 1990. Leptospirosis in Barbados. A clinical study. West Indian Medical Journal, 39(1), pp.27–34. | N | N | N | N | N | Y | N | N | On ICU. Same cohort as "Edwards, C.N. et al., 1988. Penicillin therapy in icteric leptospirosis. The American journal of tropical medicine and hygiene, 39(4), pp.388–390. " |  |  |
| Edwards, C.N. et al., 1988. Penicillin therapy in icteric leptospirosis. The American journal of tropical medicine and hygiene, 39(4), pp.388–390. | N | N | N | N | N | Y | Y | N | "All patients in our series received aggressive supportive care and early application of peritoneal dialysis when indicated." Same cohort as "Edwards, CN, Nicholson, GD, Hassell, T., 1990. Leptospirosis in Barbados. A clinical study. West Indian Medical JournaI, 39(1), pp.27–34." |  |  |
| Esen, S. et al., 2004. Impact of clinical and laboratory findings on prognosis in leptospirosis. Swiss medical weekly, 134(23-24), pp.347–52. | N | N | Y | N | N | N | ? | N | All treated with antibiotics |  |  |
| Everard, C.O.R. et al., 1995. A twelve-year study of leptospirosis in Barbados. European journal of epidemiology, 11, pp.311–320. | N | N | ? | N | Y | N | N | N | No information on treatment. |  |  |
| Flannery, B. et al., 2001. Referral Pattern of Leptospirosis Cases During a Large Urban Epidemic of Dengue. Am. J. Trop. Med. Hyg.., 65(5), pp.657–663. | N | N | Y | N | N | N | 17% dialysed | N | Patients all treated with antibiotics. |  |  |
| Fuzi, K. & Kiszel, J., 1961. Investigations on Leptospirosis in the Province of Tapio. Nepegeszsegugy, 42(6), pp.184–192. | N | N | ? | N | Y | N | N | N | No information on treatment or antibiotics. |  |  |
| Gancheva, G.I., 2013. Brief communication Leptospirosis in elderly patients. The Brazilian journal of infectious diseases, 17(5), pp.592–595. | N | N | Y | N | N | N | Y (3/15 dialysed) | N | Patients treated with antibiotics |  |  |
| Garnier M, Reilly. Les réactions méningés au cours de la spirochétose ictériènge. Comptes Rendus des Seances la Soc Biol Ses Fil Paris. 1917;80:446. | N | Y | N | N | N | N | N | N | No clear diagnosis for 11 patients in the series. |  |  |
| Gauld, W.R., 1947. An Outbreak of Weil’s Disease. The Lancet, 1((6)), pp.216–217. | N | Y | Y (7/13) | Y | N | N | N | N | Poor diagnosis in 4/13 patients and 7/13 patients treated with antibiotics or immune serum. |  |  |
| Greene, C., 1940. Liver and Biliary Tract. Arch. Int. Med, 65, p.847. | N | N | N | N | Y | N | N | N | Review. No clear information on Leptospirosis. |  |  |
| Gsell O. Leptospirosis Pomona, die Schweinehuterkrankheit. Schweiz Med Wochenschr. 1946;76(12):237. | N | N | Y | N | N | N | N | N | Penicillin used to treat severe cases but no record of which cases treated. |  |  |
| Guerrier, G. & D’Ortenzio, E., 2013. The Jarisch-Herxheimer reaction in leptospirosis: a systematic review. PloS one, 8(3), p.e59266. | N | N | Y | N | N | N | N | N | All treated with antibiotics |  |  |
| Gulland, L., 1924. Spirochaetosis Icterohaemorrhagica in East Lothian. BMJ, 1(3295), pp.313–4. | N | Y | N | N | Y | N | N | N | No clear diagnosis or isolation of leptospires |  |  |
| Haslé G, Toullec F, Vaucel M. Spirocétose ictétigène au Tonkin. Bull Soc Pathol Exot Filiales. 1935;28:551. | N | N | N | Y | N | N | N | N | 3 patients. |  |  |
| Heath, C.W.J., Alexander, A.D. & Galton, M.M., 1965. Leptospirosis in the United States. New England Journal of Medicine, 273(16), p.857. | N | N | Y (237/302) | N | Y | N | N | N | 65/302 patients untreated but no separate outcome for this untreated cohort. |  |  |
| Heringman EC, Phillips JH. Weil’s Disease. N Engl J Med. Massachusetts Medical Society; 1947 Sep 25;237(13):471–5. | N | N | N | N | N | Y | N | N | Cohort repeated in “Molner JG, Meyer KF, Raskin HA. Leptospiral infections, A survey. J Am Med Assoc. 1948;136(12):814–8.” |  |  |
| Hernández, M.S. et al., 1999. Leptospirosis en niños de la Provincia de Ciego de Ávila , Cuba. Revista da Sociedade Brasileira de Medicina Tropical, 32(2), pp.145–150.(Portuguese) | N | N | ? | N | Y | N | N | N | No information on treatment |  |  |
| Herrmann-Storck, C. et al., 2010. Severe leptospirosis in hospitalized patients, Guadeloupe. Emerging infectious diseases, 16(2), pp.331–4. | N | N | Y | N | N | N | Y | N | Treated with antibiotics. |  |  |
| Hubener E. Weilsche Krankheit, Ruckfallfieber, bilioses typhoid. Dtsch Medizinische Wochenschrift. 1917;2:1291. | N | N | N | Y | Y | N | N | N | No clear patient cohort |  |  |
| Houli, J., Da Rosa Santos, O. & Panza, M., 1970. Leptospiroses and renal manifestations. A review of 120 cases. <Manifestacoes renais na leptospirose. Revisao de 120 casos.>. Folha Medica, 60(4), pp.365–81. | N | N | ? | N | Y | N | Y | N | 115 patients with 23 deaths. No information on treatment with antibiotics. 3 patients received peritoneal dialysis. |  |  |
| Hutchison, J.H. et al., 1946. Outbreak of Weil’s Disease in the British Army in Italy. British medical journal, 1(4437), pp.81–3 | N | N | Y | Y | N | N | N | N | 17 patients but 9 treated (3 serum and 6 antibiotics) |  |  |
| Ido Y, Ito H, Wani H. Spirochaeta Hebdomadis, the causative agent of Seven day fever (Nanukayami). J Exp Med. 1919;29:199. | N | N | N | N | Y | N | N | N | No clinical information on information on patient outcome. |  |  |
| Inada R. The clinical aspects of spirocaetosis icetrohaemorrhagica or Weil’s disease. J Exp Med. 1917;26:355. | N | N | N | Y | Y | N | N | N | No clear patient cohort. |  |  |
| Inada, R. Ido, Y. Hoki, R. Kaneko, R. Ito, H., 1916. The etiology, mode of infection, and specific therapy of Weil’s Disease (spirochaetosis Icterohaemotthagica). Journal of Experimental Medicine, pp.377–402. | N | N | N | Y | Y | N | N | N | No patient cohort with clear diagnosis. |  |  |
| Inada, R., 1922. Prophylaxis and Serum Treatment of Spiro-chaetosis icterohaemorrhagica. Japan Medical World, 2(7), p.189. | N | Y | N | N | N | N | N | N | 72 cases and 22 deaths pre-serum treatment. No clear record of diagnosis. |  |  |
| Ittyachen AM, Krishnapillai TV, Nair MC, R.A., 2007. Retrospective study of severe cases of leptospirosis admitted in the intensive care unit. Journal of Postgraduate Medicine, 53(4). | N | N | Y | N | N | N | Y | N | All patients treated with antibiotics and on ICU |  |  |
| Jansen, A. et al., 2007. Sex differences in clinical leptospirosis in Germany: 1997-2005. Clinical infectious diseases: an official publication of the Infectious Diseases Society of America, 44(9), pp.e69–72. | N | N | ? | N | Y | N | ? | N | No information on treatment or dialysis/ICU |  |  |
| Jitta, N., 1934. Weil’s Disease in Holland. Bulletin de l’Office International d'Hygiene Publique, 26, pp.688–9. | N | Y | N | N | N | N | N | N | Serological diagnosis only for one half of the cases. 134 cases with a mortality of 7.5% |  |  |
| Johnson, D.W., 1950. The Australian Leptospiroses. The Medical journal of Australia, 2, p.724. | N | N | Y (30/188) | N | Y | N | N | N | 30/168 treated. No information on untreated cohort. 4 deaths in 168 patients. |  |  |
| Jorge, R., 1932. A Water-borne Epidemic of Haemorrhagic Jaundice at Lisbon. Nosology, Bacteriology and Epidemiology. Bulletin de l’Office International d'Hygiene Publique, 24, pp.88–117. | N | Y | N | N | N | N | N | N | 31/126 deaths (24.6%). No confirmed diagnosis for all patients. Only a few cases diagnosed through animal inoculation (2) or serology (10 convalescent serum examined) |  |  |
| Kathe, J., 1942. Jaundice and Mortality in Mud-Field Fever. Klinische Wochenschrift, 21(36), pp.787–790. | N | N | N | Y | Y | N | N | N | Small cohort of patients. Review of disease (German) |  |  |
| Kathe, J., 1928. The So-called Slime Fever in 1926 and 1927. Notes on the Symptomatology, Epidemiology, Pathology and Aetiology. Zentralblatt fur Bakteriologie, Parasitenkunde, Infektionskrankheiten und Hygiene, 109(5/6), pp.284–310. | N | Y | N | N | Y | N | N | N | No clear laboratory diagnosis for all patients (German) |  |  |
| Katz, A.R. et al., 2001. Assessment of the Clinical Presentation and Treatment of 353 Cases of Laboratory-Confirmed Leptospirosis in Hawaii, 1974-1998. Clinical Infectious Diseases, 33, p.1834. | N | N | Y (327/353) | N | Y | N | N | N | No information on outcome for untreated cohort. Noted to have a non-statistically significant longer duration of fever than treated cohort, however. |  |  |
| Ko, A.I. et al., 1999. Urban epidemic of severe leptospirosis in Brazil. Lancet, 354(9181), pp.820–825. | N | Y | Y | N | N | N | Y (26%) | N | All treated with Antibiotics. Significant proportion received dialysis |  |  |
| Korner. Ueber eine Epidemie von Weilscher Krankheit. Dtsch Medizinische Wochenschrift. 1925;19:772. | N | Y | N | N | N | N | N | N | No laboratory diagnosis for patients. |  |  |
| Korthof G. Experimentelles Schlammfieber beim Menschen. Zentralblatt fur Bakteriol. 1932;124(2):429. | N | N | N | Y | N | N | N | N | Only 9 patients successfully infected |  |  |
| Kotorii, S., 1935. Zur Klinik der sogenannten Hasamiyami. klinische wochenschrift jahrgang, p.1147. | N | Y | N | N | N | N | N | N | No laboratory diagnosis for patients. Historical review. (German) |  |  |
| Kouwenaar, W., 1923. Over een epidemie van koortsen zonder en met icterus, veroozaakt door een leptospira. Geneeskundig Tijdschrift voor Nederlandsche-Indie, p.225. | N | Y (43/164) | N | N | N | N | N | N | Only 43/164 patients had positive identification of leptospira in series. |  |  |
| Kramer P. Weil’s Disease in Rotterdam. Ned Tidjschrift voor Geneeskd. 1932;76:4296–303. | N | N | N | N | N | Y | N | N | Repeated cohort “Kramer P. Weil’s Disease in Rotterdam. Ned Tidjschrift voor Geneeskd. 1932;76: 4296–303.” |  |  |
| Kuriakose, M., Eapen, C.K. & Paul, R., 1997. Leptospirosis in Kolenchery, Kerala, India: Epidemiology, prevalent local serogroups and serovars and a new serovar. European journal of epidemiology, 13, pp.691–697. | N | N | ? | N | N | N | Y (16 patients) | N | No information on antibiotic treatment |  |  |
| Larson, C., 1941. Weil’s Disease: A report of 51 cases occurring in Puerto Rico and the United States. Public Health Reports, 56, pp.1650–6. | N | Y | N | N | Y | N | N | N | No clear laboratory for all patients. No information on outcome. |  |  |
| Lau, C.L. et al., 2012. Leptospirosis in American Samoa 2010: epidemiology, environmental drivers, and the management of emergence. The American journal of tropical medicine and hygiene, 86(2), pp.309–19. | N | N | N | N | N | N | N | Y | Sero-prevalence study |  |  |
| De Lavergne V, Accoyer H. La spirochétose méningée pure, considérations épid´´miologiques et pathogéniques. Rev d’Hygiene Médicine Préventative. 1937;59(5):339. | N | N | N | Y | N | N | N | N | Only 3 patients in case series. |  |  |
| Lecour, H. et al., 1989. Human Leptospirosis. A review of 50 cases. Infection, 17(1), p.8. | N | N | Y (40/50) | N | N | N | (11/31) | N | Patients treated with antibiotics or received haemodialysis if unwell. |  |  |
| Lefebvre Des Noettes, R.A., Seigneuric, C. & Kolochine-Erber, B., 1950. A contribution to the study of leptospirosis due to Leptospira grippo-typhosa, marsh and mud fever. A spring epidemic of 77 cases among Army personnel. 58(67), pp.1189–1192. | N | N | Y | N | N | N | N | N | 66 confirmed cases. (French) Antibiotics "Given to those who gave permission" Number treated not included but cohort excluded. |  |  |
| Léger M. Spirochétose ictéro-hémorragique à la Guadeloupe. Bull Soc Pathol Exot Filiales. 1932;25:304. | N | N | N | Y | N | N | N | N | 2 cases only. |  |  |
| Lester BS, Denison GA, Posey LC. Weil’s Disease: A clinical and epidemiological report of fourteen cases. J Am Med Assoc. 1942;35(4):325. | N | N | N | N | N | Y | N | N | Cohort repeated in “ Molner JG, Meyer KF, Raskin HA. Leptospiral infections, A survey. J Am Med Assoc. 1948;136(12):814–8.” |  |  |
| Lopes, A.A. et al., 2004. Comparative study of the in-hospital case-fatality rate of leptospirosis between paediatric and adult patients of different age groups. Revista do Instituto de Medicina Tropical de São Paulo, 46(1), pp.19–24. | N | N | Y | N | Y | N | Y | N | No mortality data for untreated cohort |  |  |
| Lorando NJ. Étude critique sur l’épidémie de l'ile de Syra. Dengue et spirochétose. Bull Soc Pathol Exot Filiales. 1932;25:552. | N | Y | N | N | N | N | N | N | No clear diagnosis of outbreak. |  |  |
| Mackay-Dick, J. & Robinson, J.F., 1957. Penicillin in the Treatment of 84 Cases of Leptospirosis in Malaya. J R Army Med Corps., 103(4), pp.186–97. | N | N | Y | N | N | N | N | N | All 84 patients treated. |  |  |
| Mailloux, M., 1965. Sur les sources de contamination par les Leptospires en Afrique du Nord. Zeitschrift fur Tropenmedizin und Parasitologie, 16(3), p.291–. | N | N | N | N | Y | N | N | N | Not enough information on patient cohort. Review. (French). |  |  |
| Mailloux, A., 1980. Human leptospiroses in overseas French departments: Ten years of immunological diagnosis. Bulletin de la Societe de Pathologie Exotique et de ses Filiales, 73(3), pp.229–238. | N | N | ? | N | Y | N | N | N | No information on antibiotic treatment. Mortality (35/405) |  |  |
| Marotfo, P.C.F. et al., 1997. Outcome of Leptospirosis in Children. Am. J. Trop. Med. Hyg., 56(3), pp.5–8. | N | N | N | N | N | N | Y | N | 2 patients received dialysis but unsure of which. Unable to include. |  |  |
| Mayxay, M. et al., 2013. Causes of non-malarial fever in Laos: a prospective study. The lancet global health, 1(3), pp.e46–e54. | N | N | Y | N | Y | N | N | N | No information on outcome or treatment outcomes |  |  |
| McCrumb FR, Stockard JL, Robinson CR, Turner LH. Leptospirosis in Malaya. Am J Trop Med Hyg. 1957;6:238–56. | N | N | Y | N | N | N | N | N | Some military patients treated with penicillin and some civilian with terramycin. Outcome of untreated patients not specified. |  |  |
| Mendoza, M.T. et al., 2013. Clinical profile of patients diagnosed with leptospirosis after a typhoon: a multicentre study. The Southeast Asian journal of tropical medicine and public health, 44(6), pp.1021–1035. | N | N | Y | N | N | N | Y | N | 10% treated on ICU and unclear treatment information |  |  |
| Mochtar A. Over het voorkomen van de ziekte van Weil te Semarang en omstreken. Geneeskd Tijdschr voor Ned. 1933;1182. | N | Y | N | Y | N | N | N | N | 9 patients. No laboratory diagnosis for all patients. |  |  |
| Morrissey, G., 1934. The occurrence of Leptospirosis (Weil’s Disease) in Australia. The Medical journal of Australia, 2, pp.496–7. | N | Y | N | N | Y | Y | N | N | Patients not all laboratory diagnosis. Repeated Cohort in (Cotter, T.J., 1936. Weil’s Disease in North Queensland. BMJ, Supplement, pp.51–56.) |  |  |
| Moura, L.V. et al., 1996. Acute nephritic failure in hospitalization patients with a leptospirosis diagnosis. Revista Brasileira de Medicina, 53(5), p.347. | N | N | ? | N | N | N | Y | N | 20% required peritoneal dialysis. |  |  |
| Niwattayakul, K. et al., 2002. Leptospirosis in north-eastern Thailand: hypotension and complications. Southeast Asian Journal of Tropical Medicine and Public Health, 33(1), pp.155–160. | N | N | Y | N | N | N | N | N | Treated with antibiotics. |  |  |
| Noguchi, H., 1922. Research on Yellow Fever. Comparative Aetiological, Pathological, and Epidemiological Considerations; Prevention and Treatment. The Lancet, 199(5155), pp.1185–1191. | N | / | / | / | Y | / | / | / | Patients with yellow fever |  |  |
| Nolf P, Firket J. Observations cliniques sur 100 cas de spirochétose ictéro-hémorragique. Arch Médicales Belges. 1918;71:380. | N | Y | N | N | N | N | N | N | No laboratory diagnosis for cases. Clinical diagnosis alone. |  |  |
| Noronha de Miranda, R., 1946. Doenca de Weil no parana. Revista Médica do Paraná, 15(6), pp.229–234. | N | Y | N | N | Y | N | N | N | No clear diagnosis or all patients |  |  |
| De Oliveira, TVdS., Pinheiro Marinho, D, Neto, CC, Kligerman, D., 2012. Climate variables, living conditions and the health of the population: leptospirosis in the city of Rio de Janeiro from 1996 to 2009. Ciencia e Saude Coletiva, 17(6), p.1576. | N | N | ? | N | Y | N | ? | N | No information on diagnostics or treatment |  |  |
| Ostertag, H., 1950. Leptospirosis icterohaemorrhagica in Bulgaria. <Leptospirosis icterohaemorrhagica in Bulgarien.>. Zeitschrift fur Hygiene und Infektionskrankheiten, 131(5), pp.482–500. | N | Y | N | N | N | N | N | N | 8/56 deaths. No clear diagnosis for all patients. |  |  |
| Padilla Perez, O, Toledo Vila, CHJ, Garcia, IV, Rodriguez Avila, I., 1998. Comportamiento de la mortalidad por leptospirosis en Cuba, 1987-1993. , 50(1), pp.61–65. | N | N | ? | N | Y | N | ? | N | No information on treatment or dialysis/ICU |  |  |
| Paganin, F. et al., 2007. Leptospirosis in Reunion Island (Indian Ocean): analysis of factors associated with severity in 147 confirmed cases. Intensive care medicine, 33(11), pp.1959–66. | N | N | N | N | N | N | Y (80/147) | N |  |  |  |
| Panaphut, T. et al., 2003. Ceftriaxone compared with sodium penicillin G for treatment of severe leptospirosis. Clinical Infectious Diseases, 36(12), pp.1507–1513. | N | N | Y | N | N | N | ? | N | Patients all treated with antibiotics. |  |  |
| Pappachan, M.J. et al., 2004. Risk factors for mortality in patients with leptospirosis during an epidemic in northern Kerala. National Medical Journal of India, 17(5), pp.240–2. | N | N | Y | N | N | N | N | N | Patients all treated with antibiotics. |  |  |
| Park, S.K. et al., 1989. Leptospirosis in Chonbuk Province of Korea in 1987: A Study of 93 Patients. Am. J. Trop. Med. Hyg., 41(3), p.345. | N | N | Y | N | N | N | N | N | Patients treated with antibiotics. |  |  |
| Pérez Carril, R. & Kozmin-Sokolov, B., 1967. Leptospirosis in Oriente Norte province (Cuba). II. Clinico-microbiological study and isolation of leptospires. Bol. Hig. Epidem, 5(2), pp.137–140. | N | N | N | N | Y | N | N | N | Review of Leptospirosis with no clear patient cohort. |  |  |
| Perrocheau, A. & Perolat, P., 1997. Epidemiology of leptospirosis in New Caledonia (South Pacific): A one-year survey. European journal of epidemiology, 167, pp.161–167. | N | N | Y | N | Y | N | N | N | Mostly treated. No separate information on untreated cohort |  |  |
| Pertuiset, E. et al., 1988. Clinical features and prognosis of leptospirosis (Weil’s disease) in adults. A study of 249 cases in La Reunion. Revue de Medecine Interne, 9(5), p.487. | N | N | Y (155/241) | N | Y | N | Y (56/249) | N | Significant proportion received dialysis. Mortality 7% amongst 86 patients who did not receive antibiotics. No record on whether these patients received antibiotics however. |  |  |
| Pertzelan, A, Pruzanski, W., 1963. Leptospira Canicola Infection: Report of 81 Cases and Review of the Literature. Am. J. Trop. Med. Hyg., 12(1), pp.75–81. | N | Y | Y | N | Y | N | N | N | Treated with antibiotic (unspecified) and diagnosis not accurate for all cases. |  |  |
| Pettit, A., 1926. La sérothérapie des spirochétoses ictéro-hémorragiques et de la poliomyélite. Le progrés médical, 8, pp.279–289. | N | Y | Y | Y | Y | N | N | N | Treated with serum. No cohort. |  |  |
| Pinn, T.G., 1992. Leptospirosis in the Seychelles. Medical Journal of Australia, 156(3), p.163. | N | N | Y | N | N | N | N | N | All patients treated with antibiotics. |  |  |
| Ragiot C, Delbove P. Spirochétose ictétigène en Cochinchine. Bull Soc Pathol Exot Filiales. 1934;27:347. | N | N | N | Y | N | N | N | N | 2 cases only. |  |  |
| Ragnaud, J.M. et al., 1993. Epidemiologic, clinical, biological, and evolutionary aspects of leptospirosis: 30 case reports in Aquitaine. Revue de Medecine Interne, 14(6), p.425. | N | N | Y (22/30) | N | N | N | Y (7/30) | N | Majority treated or received ICU treatment. |  |  |
| Rajapakse, S., Rodrigo, C. & Haniffa, R., 2010. Developing a clinically relevant classification to predict mortality in severe leptospirosis. Journal of emergencies, trauma, and shock, 3(3), pp.213–9. | N | N | N | N | Y | N | N | N | Review and no untreated cohort. |  |  |
| Ramachandran, S. et al., 1976. Patterns of acute renal failure in leptospirosis. Journal of Tropical Medicine and Hygiene, 79(9), p.158. | N | N | ? | N | Y | N | ? | N | No information on treatment on renal replacement therapy. |  |  |
| Ramachandran, S., Rajapakse, C.N.A. & Perera, M.V.F., 1974. Changing Patterns in Leptospirosis in Sri Lanka. Ceylon Medical Journal, December, pp.142–149. | N | N | ? | N | Y | N | N | N | No information on antibiotic treatment. |  |  |
| Rankov, M., 1955. Uber eine Trinkwasserepidemie von Weilscher Krankheit. Zeitschrift fur Hygiene und Infektionskrankheiten, 140(S), pp.556–572. | N | Y | N | N | Y | N | N | N | 390 infected patients 2% mortality. No clear diagnosis for all patients |  |  |
| Raoult, D, Jeandel, P, Mailloux, M, Rougier, Y., 1983. Thrombocytopenia and Renal Failure in Leptospirosis. Am. J. Trop. Med. Hyg., 32(6), p.1983. | N | N | ? | N | Y | N | N | N | No information on treatment. |  |  |
| Reiter, H., 1934. La Maladie de Weil en Allemagne. Bulletin de l’Office International d'Hygiene Publique, 26, pp.1747–8. | N | Y | N | N | Y | N | N | N | No Clear information on Patient outcome or diagnostics. |  |  |
| Renault, P. et al., 2011. Surveillance epidemiologique de la leptospirose a la Reunion, 2004-2008: possible impact de l’epidemie de chikungunya sur la letalite de la leptospirose. Bull. Soc. Pathol. Exot, 104(2), pp.148–152. | N | Y | ? | N | Y | N | N | N | No clear information on treatment |  |  |
| Renaux E. Note sur la spirochétose ictéro-hémorragique. C R Seances Soc Biol Fil. 1916;79:947. | N | N | N | N | N | Y | N | N | Cohort repeated in “ Wilmaers L, Renaux E. Quarante-sept cas de spirochetose icterohemorragique; etude clinique et notes de laboratoire. Arch Med Belges. 1917;70:115.” |  |  |
| Robertson, K., 1946. Weil’s Disease: A rare condition? British Medical Journal, 2(4482), pp.810–813. | N | N | N | N | N | Y | N | N | Patient cohort repeated in "Broom, J.C., and Alston, J.M., “Weil’s Disease” Lancet 2, 96 1948". |  |  |
| Roche, G., Gerard, A. & Schaefer, M., 1982. Current aspects in leptospirosis. (26 cases). Annales Medicales de Nancy et de l’Est, pp.529–539. | N | N | Y (23/26) | N | N | N | N | N | Majority of patients treated with antibiotics. |  |  |
| Romijn P. De ziekte van weil te Dordrecht. Ned Tidjschrift voor Geneeskd. 1932;IV:5832. | N | N | N | N | N | Y | N | N | Patient cohort repeated in “Walch-Sorgdrager B. Leptospiroses. Bull Heal Organ (League Nations). 1939;8:143–386.” |  |  |
| Sanders, E.J. et al., 1999. Increase of leptospirosis in dengue-negative patients after a hurricane in Puerto Rico in 1996 [correction of 1966]. The American journal of tropical medicine and hygiene, 61(3), pp.399–404. | N | N | ? | N | Y | N | ? | N | No information on treatment |  |  |
| Sapian, M. et al., 2012. Outbreak of Melioidosis and Leptospirosis Co-infection Following a Rescue Operation. Medical Journal of Malaysia, 67(3), pp.293–297. | N | N | ? | Y | Y | N | ? | N | Only 4 patients with Leptospirosis. No information on treatment |  |  |
| Sarkar, U. et al., 2002. Population-based case-control investigation of risk factors for leptospirosis during an urban epidemic. The American journal of tropical medicine and hygiene, 66(5), pp.605–610. | N | N | ? | N | Y | N | Y | N | No information on antibiotic treatment. Unwell patients treated n ICU. |  |  |
| Schuffner, W., 1934. Recent Work on Leptospirosis. Transactions of the Royal Society of Tropical Medicine and Hygiene, 28(1), pp.7–31. | N | N | N | N | N | Y | N | N | Repeated Cohort: “Leptospiroses Walch-Sorgdrager B., Bull. Health Organization (League of Nations) 8:144-346.  1939. “ |  |  |
| Schuffner, W.A.P., 1942. A Critical Study on the Occurrence of Jaundice and Death in “Mud Fever.” Klinische Wochenschrift, 21(36), pp.787–90. | N | Y | N | N | Y | N | N | N | Review. No Clear information on diagnostics and outcomes of cohorts included. |  |  |
| Schuffner WAP. Weil’s Disease in Holland. Ned Tidjschrift voor Geneeskd. 1932;76:5584–56. | N | N | N | N | N | Y | N | N | Repeated patient cohort: “Schuffner W. Weil’s Disease in the Maritime Countries. Dtsch Medizinische Wochenschrift. 1941;67(15):393–9.” |  |  |
| Schwetz J, Kadener. Sur une épidémie mystérieuse observée en 1932 parmi les Européens de Stanleyville et en relation avec un bassin de natation. Bull Soc Pathol Exot Filiales. 1934;27:354. | N | Y | N | N | N | N | N | N | No clear diagnosis for all patients. |  |  |
| Segura, E.R. et al., 2005. Clinical spectrum of pulmonary involvement in leptospirosis in a region of endemicity, with quantification of leptospiral burden. Clinical infectious diseases: an official publication of the Infectious Diseases Society of America, 40(3), pp.343–51. | N | N | ? | N | Y | N | ? | N | No information on treatment or ICU care. |  |  |
| Seguro, A., Lomar, A. V & Rocha, A.S., 1990. Acute renal failure of leptospirosis: Non-oliguric and hypokalemic forms. Nephron, 55(2), p.146. | N | N | N | N | N | N | Y (21/56) | N | High proportion of patients treated with dialysis. No information on antibiotic treatment. |  |  |
| Sethi, S. et al., 2010. Increasing trends of leptospirosis in Northern India: A clinico-epidemiological study. PLoS Neglected Tropical Diseases, 4(1), p.e579. | N | N | Y (86/86) | N | N | N | ? | N | Patients all treated. |  |  |
| Silva Júnior, G.B. et al., 2011. RIFLE and Acute Kidney Injury Network classifications predict mortality in leptospirosis-associated acute kidney injury. Nephrology (Carlton, Vic.), 16(3), pp.269–76. | N | N | Y | N | N | N | Y | N | Some patients treated with antibiotics and on ICU. |  |  |
| Smith, J., 1941. The Treatment of Weil’s Disease. The Medical Press and Circular, 206(4), pp.87–89. | N | N | Y | Y | N | Y | N | N | No clear cohort. Repeated information on previous case series “Davidson, L.S.P. & Smith, J., 1939. Weil’s disease in the North-East of Scotland. BMJ, 2(4110), pp.753–757.” |  |  |
| Smits, E., 1932. Ziekte van weil op tarakan (borneo). Geneeskundig Tijdschrift voor Nederlandsche-Indie, p.284. | N | Y | N | N | N | N | N | N | 26 patients  and 3 deaths. Majority diagnosed clinically, although no information on symptoms, as "clear cases". Excluded as only doubtful cases confirmed through inoculation of guinea pigs and clinical diagnoses are excluded. |  |  |
| Spichler, A.S. et al., 2008. Predictors of lethality in severe leptospirosis in urban Brazil. The American journal of tropical medicine and hygiene, 79(6), pp.911–914. | N | N | ? | N | N | N | ? | N | No information on treatment |  |  |
| Spichler, A. et al., 2012. Comparative analysis of severe pediatric and adult leptospirosis in Sao Paulo, Brazil. The American journal of tropical medicine and hygiene, 86(2), pp.306–8. | N | N | ? | N | Y | N | ? | N | No information on antibiotic treatment |  |  |
| Stiles, W.W. & Sawyer, W.A., 1942. Leptospiral infection as an occupational hazard. Journal of the American Medical Association, 118, p.34. | N | N | N | N | N | N | N | N | Repeated cohort of Published American Case reports of Leptospirosis in "Molner, J.G., Meyer, K.F. & Raskin, H.A., 1948. Leptospiral infections, A survey. Journal of the American Medical Association, 136(12), pp.814–818." |  |  |
| Strasburger J. Zur klinik der Weilschen krankeit. Dtsch Arch für Klin Medizin. 1918;125:108. | N | Y | N | N | Y | N | N | N | No clear diagnosis for 26 patients. |  |  |
| Stokes, A., Ryle, J. & Tytler, W., 1917. Weil’s Disease (Spirochaetosis Ictero-haemorrhagica) in the British Army in Flanders. The Lancet, 189(4874), pp.142–153. | N | Y | N | N | N | N | N | N | No confirmed diagnosis. Majority of patients diagnosed clinically. |  |  |
| Strobel, M. et al., 1992. Leptospirosis in Guadeloupe (French West Indies). Clinical, biological and epidemiological aspects. Medecine et Maladies Infectieuses, 22(6-7), p.648. | N | N | ? | N | Y | N | N | N | No information on antibiotic treatment. |  |  |
| Suputtamongkol, Y. et al., 2004. An open, randomized, controlled trial of penicillin, doxycycline, and cefotaxime for patients with severe leptospirosis. Clinical infectious diseases: an official publication of the Infectious Diseases Society of America, 39(10), pp.1417–1424. | N | N | Y | N | N | N | ? | N | Patients all treated with antibiotics. |  |  |
| Tarassoff, S., 1934. Brief History of Infective Jaundice and Leptospirosis in Soviet Russia. Bulletin de l’Office International d'Hygiene Publique, 26, pp.690–700. | N | Y | N | N | Y | N | N | N | No clear diagnosis for patient cohorts described. |  |  |
| Tartaglia, P., 1959. Leptospiroses in Dalmatia. <O leptospirozama u Dalmaciji.>. Lijecnicki Vjesnik, 81(1-2), pp.16–20. | N | N | Y | N | N | N | N | N | "Antibiotic  therapy introduced despite divided opinions on their effectiveness" No record of number treated (Bosnian). |  |  |
| Thorner W. Zur klinik des ikterus onfektiosus. Dtsch Medizinische Wochenschrift. 1917;2:1071. | N | Y | N | N | Y | N | N | N | No clear diagnosis for patients. |  |  |
| Trimble, A.P., 1957. Clinical Aspects of Leptospirosis in Malaya. Proc. R. Soc. Med, 50(2), pp.125–8. | N | N | ? | N | N | N | N | N | Cohort of the patients treated but no specific information on treatment and outcome. Overall 236 patients with 8 fatalities. |  |  |
| Cochez P, Fichet. Nouvelles observations de Spirochétose méningé anictérique. Presse Med. 1933;32:647. | N | N | N | Y | N | N | N | N | Single case. |  |  |
| Turner, L.H. et al., 1959. Acute febrile illnesses in Malaya: Leptospirosis. The Medical Journal of Malaya, 13(2), pp.83–98. | N | N | Y | N | N | N | N | N | Review with later cohorts treated. Earlier cohort mentioned in article is included in review "Fletcher, W., 1927. Leptospirosis, tsutsugamushi disease and tropical typhus. Transactions of the Royal Society of Tropical Medicine and Hygiene, 21, p.265." |  |  |
| Turner, L.H., 1967. Leptospirosis I. Transactions of the Royal Society of Tropical Medicine and Hygiene, 61(6), pp.842–855. | N | N | N | Y | Y | N | N | N | Review. No patient cohort. |  |  |
| Valassopoulo A. A propos de la spirochétose ictérohémorragique. Bull la Société médicale des Hosp. 1917;41:920. | N | Y | N | N | N | N | N | N | No clear laboratory diagnosis for patients. |  |  |
| Varfolomeeva, A.A., 1957. Epidemiology and aetiology of an outbreak of Leptospirosis. Journal of Microbiology, Epidemiology and Immunobiology, 28(1), pp.38–43. | N | Y | N | N | Y | N | N | N | No clear information on patient numbers, outcome, or diagnosis. |  |  |
| Vaucel M, Soulier R. Sur l’existence d'un foyer de leptospirose a Tuyen-Quang (Tonkin). Bull Soc Pathol Exot Filiales. 1937;30:408. | N | N | N | N | Y | N | N | N | No clear clinical information on outcome of patients. |  |  |
| Victoriano, A.F.B. et al., 2009. Leptospirosis in the Asia Pacific region. BMC infectious diseases, 9, p.147. | N | N | N | N | Y | N | N | N | Review. Not adequate information on patient cohorts |  |  |
| Vijayachari, P. et al., 2004. Leptospirosis among schoolchildren of the Andaman & Nicobar Islands, India: low levels of morbidity and mortality among pre-exposed children during an epidemic. Epidemiology and Infection, 132(6), pp.1115–1120. | N | N | ? | N | Y | N | ? | N | No information on treatment |  |  |
| Watt G, Padre LP, Tuazon ML, Calubaquib C, Santiago E, Ranoa CP, et al. Placebo-controlled trial of intravenous penicillin for severe and late leptospirosis. Lancet 1988; 1 (8583): 433–5. | N | N | Y | N | N | N | N | N | Many patients treated with antibiotic pre admission onto the "control" cohort. |  |  |
| Yang, H.-Y. et al., 2012. Early identification of leptospirosis as an ignored cause of multiple organ dysfunction syndrome. Shock (Augusta, Ga.), 38(1), pp.24–9. | N | N | Y | N | N | N | Y | N | Treated with antibiotics. |  |  |
| Yersin, C. et al., 2000. Pulmonary haemorrhage as a predominant cause of death in leptospirosis in Seychelles. Transactions of the Royal Society of Tropical Medicine and Hygiene, 94, pp.71–76. | N | N | Yes (75/75) | N | N | N | Y (8/75) | N | Treated with antibiotics. |  |  |
| Zuelzer, M., 1936. Biologie und epidemiologie der Weilschen Krankheit. Zentralblatt fur Bakteriologie, 137, p.189. | N | N | N | N | Y | N | N | N | Review of diagnostic techniques (German). |  |  |
