# Supplementary material for: A Systematic Review of the Mortality from Untreated Leptospirosis
Source: PLoS Negl Trop Dis. 2015 Jun 25;9(6):e0003866. doi: 10.1371/journal.pntd.0003866 (PMC4482028; doi:10.1371/journal.pntd.0003866)
Supplement: S5 Table — (DOCX) [file pntd.0003866.s012.docx]

### Supplementary Table 5: Bias within each patient series. Red reports high bias, yellow medium bias and green low bias. (see scoring bias color coding in Suppl. Table 2.)

Patient Selection

Diagnostic Test

Information Quality

Patient Outcome

| Baermann 1928 |  |  |  |  |
| --- | --- | --- | --- | --- |
| Berman 1973 |  |  |  |  |
| Borg-Petersen 1949 (1) |  |  |  |  |
| Borg-Petersen 1949 (2) |  |  |  |  |
| Borg-Petersen 1949 (3) |  |  |  |  |
| Broom 1948 |  |  |  |  |
| Broom 1951 (1) |  |  |  |  |
| Broom 1951 (2) |  |  |  |  |
| Btesh 1947 |  |  |  |  |
| Bulmer 1945 |  |  |  |  |
| Cavigneaux 1948 |  |  |  |  |
| Fairburn 1956 |  |  |  |  |
| Fairley 1934 |  |  |  |  |
| Fletcher 1928 |  |  |  |  |
| Gardner 1946 |  |  |  |  |
| Hall 1951 |  |  |  |  |
| Ido 1918 |  |  |  |  |
| Kocen 1962 |  |  |  |  |
| Kouwenaar 1925 (1) |  |  |  |  |
| Kouwenaar 1925 (2) |  |  |  |  |
| Kristensen 1935 |  |  |  |  |
| McClain 1984 |  |  |  |  |
| Minkenhof 1948 |  |  |  |  |
| Molner 1948 (1) |  |  |  |  |
| Molner 1948 (2) |  |  |  |  |
| Mulder 1931 |  |  |  |  |
| Patterson 1947 |  |  |  |  |
| Robinson 1956 |  |  |  |  |
| Rugiero 1948 |  |  |  |  |
| Russell 1958 |  |  |  |  |
| Schuffner 1941 (1) |  |  |  |  |
| Schuffner 1941 (2) |  |  |  |  |
| Senekjie 1944 |  |  |  |  |
| Slot 1932 |  |  |  |  |
| Smith 1949 |  |  |  |  |
| Swan 1938 |  |  |  |  |
| Taylor and Goyle 1930 |  |  |  |  |
| Van Riel 1939 |  |  |  |  |
| Vervoort 1923 |  |  |  |  |
| Walch Sorgdrager 1939 |  |  |  |  |
| Wilamers 1917 |  |  |  |  |
