# Supplementary material for: A Systematic Review of the Mortality from Untreated Leptospirosis
Source: PLoS Negl Trop Dis. 2015 Jun 25;9(6):e0003866. doi: 10.1371/journal.pntd.0003866 (PMC4482028; doi:10.1371/journal.pntd.0003866)
Supplement: S6 Table — (DOCX) [file pntd.0003866.s013.docx]

### Supplementary Table 6: Assessment of bias within studies

|  | **Patient Selection** | **Diagnostic Test** | **Missing Information** | **Outcome** |
| --- | --- | --- | --- | --- |
| **Study** | **Consecutive / Inappropriate Exclusions/Difficult to Diagnose Patients Excluded?** | **How Reliable is the Diagnostic Test?**  **(Grade)** | **Blinding / Loss to Follow Up / Lacks Information / Selective Outcome Reporting / Unreliable Information** |  |
| Baermann, G. B. Cent. F. Bakt 1928 | 196 patients with a confirmed diagnosis of leptospirosis from a prospective, consecutive series. 400 patients with a clinical diagnosis of leptospirosis but only 196 confirmed to have leptospirosis with laboratory methods. | 196/196 identified though culture or guinea pig inoculation and identification of leptospires. (Grade I) | No information on age or sex or duration of symptoms. No information on renal function. | Outcome recorded for all included patients (196/400) |
| Berman SJ et al. 1973. | Prospective, consecutive case series selected from all patients admitted with fever. 51 excluded due to no diagnosis. 49 patients excluded as treated. No randomisation of treatment as most unwell patients were treated. Unwell patients excluded. | (150/150) Single High titre MAT. Low specificity and sensitivity. (27/150 also culture positive). (Grade III) | No information on age. Missing information on renal function. | Recorded for all patients but 100/201 excluded due to missing information or treatment. No loss to follow up. |
| Borg-Petersen C. et al. 1949  (1) | Samples sent to national reference laboratory if suspected to be leptospirosis by local doctor. Non-consecutive cases. Retrospective review of positive cases. Cases unlikely to be representative of whole population. | (254/254) Single high titre MAT. Low sensitivity and specificity. . No record of titre required for positive diagnosis. (Grade III) | No information on length of symptoms or renal function. No information on age of patients. ? Unreliable information as relied on reported information from requesting doctor. | Only outcome of mortality and jaundice reported. Reported for all patients |
| Borg-Petersen C. et al. 1949  (2) | Samples sent to national reference laboratory if suspected as leptospirosis by local doctor. Non-consecutive cases. Retrospective review of positive cases. Cases unlikely to be representative of whole population. | (459/459) Single high titre MAT. Low sensitivity and specificity. . No record of titre required for positive diagnosis. (Grade III) | No information on length of symptoms or renal function. No information on age of patients. ? Unreliable information as relied on reported information from requesting doctor. | Only outcome of mortality and jaundice reported. Reported for all patients |
| Borg-Petersen C. et al. 1949  (3) | Samples sent to national reference laboratory if suspected leptospirosis by local doctor. Non-consecutive cases. Retrospective review of positive cases. Cases unlikely to be representative of whole population. | (95/95) Single high titre MAT. Low sensitivity and specificity. . No record of titre required for positive diagnosis. (Grade III) | No information on length of symptoms or renal function. No information on age of patients. ? Unreliable information as relied on reported information from requesting doctor. | Only outcome of mortality and jaundice reported. Reported for all patients |
| Broom, J. C. Lancet 1948 | Samples sent to national reference laboratory if suspected leptospirosis by local doctor. Retrospective review of non-consecutive cases. Unlikely to be representative of whole population. Selection bias for more unwell cohort | 195/195 single high titre MAT (>1:300). Low sensitivity and specificity. (Grade III) | No information on duration of symptoms or renal function. Incomplete information on age and sex. | Outcome missing in 81/195 patients. Clinical information on jaundice only. |
| Broom, J. C. Lancet 1951  (1) | Samples sent to national reference laboratory if suspected leptospirosis by local doctor. Retrospective. Unlikely to be representative of whole population. Selection bias for more unwell cohort. No randomisation of treatment. | (259/259) Single high titre. No record of significant titre for positive diagnosis. (Grade III) | No information on duration of symptoms or renal unction. Incomplete information on age. | 206/465 patients treated and excluded from final cohort. Outcome recorded as a percentage. |
| Broom, J. C. Lancet 1951  (2) | Samples sent to national reference laboratory if suspected leptospirosis by local doctor. Retrospective. Unlikely to be representative of whole population. Selection bias for more unwell cohort. | (70/70) Single high titre. No record of significant titre for positive diagnosis. (Grade III) | No information on duration of symptoms or renal unction. Incomplete information on age. | No patients excluded. |
| Btesh, S. TRSTMH 1947 | Long delay from illness to diagnosis in some cases. Small, retrospective summary of case reports. Unlikely to be representative of the incidence in the whole population. 2 patients treated but no randomisation of treatment. | Single high titre (13/17) and Leptospira on autopsy (4/17). Performed up to 6 months after illness. (Grade III) | Comprehensive demographic and clinical data. | 2/17 patients excluded as treated. Outcome for all included patients known. |
| Bulmer, E. BMJ 1945 | Non-randomised treatment of patients. Prospectively, non-consecutive recruitment of cohort. More unwell cohort selected. "My figures must be incomplete and cases must have been missed" Over 100 patients unwell in the outbreak but only 36 confirmed bacteriologically - difficult to diagnose patients excluded. | Single high titre for the majority. No information on significant titre. (Grade III) | No information on age, duration of illness or renal function. | 16 cases excluded as treated. Outcome known for all patients |
| Cavigneaux, C  Arch. Mal. Prof. Med. Trav. Soc. Sec. 1948 | Retrospective review of outbreak of cases in Paris. Diagnostic method unlikely to have been positive for all patients affected by the disease. Less unwell patients likely not diagnosed. | Single High Titre MAT 21/28 “definitive” 7/28 “borderline” 4/32 autopsy (Grade III) | No information on renal failure or duration of fever. | Recorded for all patients (32/32). |
| Fairburn AC Lancet 1956 | Consecutive patients recruited prospectively for RCT. Placed in treatment groups once diagnosis known. | Paired serum (31/31) (Grade I) | Incomplete information on renal function. | 52/83 patients excluded as treated. Outcome known for all patients |
| Fairley N.H.,  BMJ 1934 | Retrospective case series of non-consecutive cases confirmed as Leptospirosis. | Single high titre (>1:30) MAT. Diagnosis by single titre up to 5 years after the event in one case. (Grade III) | No information on renal function or duration of fever. | 10/10 patients included in outcome. |
| Fletcher, W TRSHTM 1928 | Prospective, consecutive cases diagnosed as leptospirosis. | 13/20 guinea pig inoculation. 13/21 positive urine microscopy 18/21 positive blood culture (Grade I) | No information on renal function. No information on age or sex | 32/32 outcome recorded |
| Gardner  Lancet 1946 | Samples sent to national reference laboratory if suspected leptospirosis by local doctor. Retrospective case series. Not consecutive series and unlikely to be representative of whole population. | Single high titre (>1:400) MAT. (Grade II) | No information on jaundice status, renal function or other clinical data. | 16 deaths recorded. Outcome not known for all cases in the series - may not have been complete record of all deaths. |
| Hall, H E Ann. Int. Med 1951 | Prospective RCT with No information if consecutive patients. Unlikely to be representative of the whole population. Treatment not randomised - Less unwell cohort in untreated group. | 7 Blood culture 3 urine culture 2 Paired rise in MAT (Grade I) | No information on age or sex | Untreated patients 12/79. Treated patients excluded. Most unwell patients treated - untreated patients less unwell. |
| Ido, Y  JEM 1918 | Prospective, non-consecutive series of patients with a presumed clinical diagnosis of seven-day fever. | All patients confirmed diagnosis through Guinea pig inoculation and identification of spirochetes. (Grade 1) | No information on age or sex or other clinical data | 3 patients excluded as no laboratory diagnosis. |
| Kocen RS.  BMJ 1962 | Prospective NRCT. Consecutive patients but some patients treated. | “Confirmed by blood culture or serology” (grade III) | No precise information on age. No clear diagnosis of renal involvement or bilirubin | 33/61 patients in untreated cohort. Penicillin treated patients excluded. |
| Kouwenaar, R, W. Far Eastern Assoc. Trop. Med. Trans. 1925  (1) | Prospective, consecutive, case series. | Confirmed through direct observation of leptospira in urine, blood or on autopsy (58/58) (Grade I) | No information on demographics of patients with age or sex. No information on duration of symptoms. No clear definition of renal failure. | Outcome reported for all 32/32 patients |
| Kouwenaar, R, W. Far Eastern Assoc. Trop. Med. Trans. 1925  (2) | Prospective, consecutive, case series. | Confirmed through direct observation of leptospira in urine, blood or on autopsy (32/32) (grade I) | No information on demographics of patients. No information on duration of symptoms. No clear definition of renal failure | Outcome reported for all 90/90 patients |
| Kristensen, B Ugeskrift fur lager 1935 | Retrospective summary of cases. "Many cases likely not diagnosed as sero-reaction failed". Likely selection bias of patients and not representative of entire population | Single high titre MAT. No record of titre (Grade III) | Missing information on duration of fever and renal status. | Outcome for all patients (19/19) |
| McClain, B L Ann. Int. Med 1984 | RCT. Patients with life threatening leptospirosis excluded from cohort. Treated patients excluded. All patients with fever investigated for leptospirosis. | 28/29 isolation of leptospires from blood or urine. 1/29 patient serological conversion only. (Grade I) | Missing information on renal function and age. | Treated patients excluded. Outcome for (15/69) untreated patients recorded. |
| Minkenhof, J. E.  Lancet 1948 | Retrospective case series. Non-consecutive cases. | Blood (3/17) MAT single titre ≥1:1000  (Grade II) | No clinical information on age, sex, renal function or other clinical data | Outcome known for all patients. |
| Molner, J G JAMA 1948  (1) | Retrospective review of cases diagnosed in Detroit. | 73/78 Single MAT (≥1:300) 5 by autopsy. (Grade II) | No information on renal function or jaundice status for all patients. No information on sex or age | Outcomes for all patients known (78/78) |
| Molner, J G JAMA 1948  (2) | Summary of case report in USA 1905-1941 | (178/178) "Confirmed diagnosis" (178/178) (Grade III) | No information on secondary outcomes. | Outcomes known for all patients  (178/178) |
| Mulder J, et al. Geneeskd Tijdschr voor Ned. 1931 | Prospective, consecutive, case series. Diagnostic method unlikely to have been positive for all patients affected by the disease. Less unwell patients likely not diagnosed. | 41/50 guinea pig inoculation.  18/50 MAT Single titre (≥1:400) (grade II) | No information on sex, renal function and duration of illness. Missing information on renal function. | Outcome known for all patients (50/50). No patients excluded. |
| Patterson, H.M. JAMA 1947 | Retrospective case series of all cases diagnosed in a Hawaiian hospital. Unsure if consecutive cases as not all patients with fever tested | (44/44) Single high titre MAT (≥1:300). Some with paired and rising titre  (Grade II) | No information on age, sex, presence of jaundice or renal status. | Outcome known for all patients. 17/61 excluded as treated with antibiotics or blood products. |
| Robinson, C.R.  JRAMC 1956 | Retrospective case series of outbreak of cases with fever. Consecutive series. | (20/23) Positive culture  (28/29) Single high titre MAT  (Titre not specified)  (Grade III) | No information on age of patients | (2/31) patients excluded as no laboratory diagnosis |
| Rugiero HR, Rev Med Cienc Afines. 1948 | Retrospective review of cases admitted during an outbreak. Diagnostic method unlikely to have been positive for all patients affected by the disease. Less unwell patients likely not diagnosed. | 12/12 single high titre MAT (Grade III) | No information on renal function or sex. No information on duration of symptoms. | Outcome known for all patients (12/12). No patients excluded |
| Russell RW.  Lancet 1958 | Prospective series of consecutive patients. Alternate patients treated/untreated. Not blinded. Some patients later excluded as diagnosed as malaria. | "Confirmed by culture or serology" 27/27. (Grade III) | Information present of all categories. | Outcome known for all untreated patients (25/52). Penicillin treatment arm excluded. |
| Schuffner, W. Deutsche Medizinische Wochenschrift; 1941 (German) | Samples sent to national reference laboratory if suspected leptospirosis by local doctor. Unlikely to be representative of whole population. | 272/272 patients confirmed by single high titre MAT. No record of significant titre. (Grade III) | No information on age or sex of patients. No information on duration of symptoms. | Outcome known for all patients (272/272) |
| Schuffner, W. Deutsche Medizinische Wochenschrift; 1941 (German) | Retrospective case series. Samples sent to national reference laboratory if suspected leptospirosis by local doctor. Unlikely to be representative of whole population. | 258/258 patients confirmed by single high titre MAT. No record of significant titre. (Grade III) | No information on age or sex of patients. No information on duration of symptoms. | Outcome known for all patients (158/158) |
| Senekjie. JAMA 1944 | Retrospective review of patients with confirmed diagnosis of leptospirosis. Serology used for the majority of patients with low sensitivity. Case series not likely to be completely representative of all patients admitted with leptospirosis. | 24/30 single high titre MAT (>1:300) 7/30 through direct visualisation of leptospires (Grade II) | No clear information on age and duration of symptoms. | No patients excluded. Outcome for all patients (30/30) known. |
| Slot, G.A.  Geneeskd Tijdschr voor Ned. 1932 (Dutch) | Retrospective case series. Serology used for the diagnosis of many patients with lower specificity. Case series not likely to representative of all patients with leptospirosis. | (3/17) Leptospirosis Culture, (7/17) Animal inoculation, (7/17) Positive agglutination (≥1:400) (Grade II) | No information on age, sex, renal function and duration of symptoms | Outcome known for all patients. No patients excluded. |
| Smith J.,  Brit J. Industr. Med 1949 | Retrospective review of patients with confirmed diagnosis of leptospirosis. Serology used for the majority of patients with low specificity. Case series not likely to be completely representative of all patients admitted with leptospirosis. Treated patients excluded who are likely to be most unwell. | 100% (214/214) high titre (<1:10) 45% (93/187) Animal inoculation (Grade (III) | No clear information on duration of fever. | Treated patients excluded. Outcome for 198/198 known. |
| Swan, W G A Newcastle Medical Journal 1938 | Retrospective review of patients with confirmed diagnosis of leptospirosis. Case series not likely to be completely representative of all patients admitted with leptospirosis. Treated patients excluded who are likely to be most unwell. | (8/30) Positive inoculation of Guinea Pig (9/30) Diagnosed at autopsy Single high titre MAT (23/30) (Grade III) | No clear information on duration of fever. | Treated patients excluded. Outcome for known for (18/18) |
| Taylor & Goyle  Ind J Med Res 1931 | Consecutive prospective case series. Patients without clear diagnosis excluded therefore non-consecutive case series. | 46/46 Identification of leptospires through blood culture, animal inoculation or urine microscopy. (Grade I) | No clear information on age or renal function. | Patients without bacteriological isolation of leptospires excluded |
| Van Riel AS Bel. Med. Trop. 1939 | Retrospective review of patients with confirmed diagnosis of leptospirosis. Serology used for the majority of patients with low sensitivity. Case series not likely to be completely representative of all patients admitted with leptospirosis. | Single high titre MAT (32/32) Unsure of positive titre. (Grade III) | No clear information on sex of patients. No information on renal function. | No patients excluded. Outcome known for all patients (32/32) |
| Vervoort H. Geneeskd Tijdschr voor Ned. 1923 (Dutch) | Prospective case series of consecutive patients admitted with fever. Diagnostic method used culture so likely to have low sensitivity. | Culture positive (90/90) (grade I) | No clear information on age or sex or duration of symptoms. No information on renal function. | No patients excluded. Outcome for all patients (90/90) |
| Walch-Sorgdrager  Bul. Health Org 1939 | Retrospective case series. Samples sent to national reference laboratory if suspected leptospirosis by local doctor. Unlikely to be representative of whole population. | (4/12) positive urine culture. (12/12) positive serology (>1:300). (Grade II) | No information on demographics or clinical data | No patients excluded. |
| Wilmaers et Renaux  Arch. Méd. Belges 1917 | Prospective consecutive case series. Patients without clear bacterial diagnosis excluded | (22/22) Isolation of leptospires. (Grade I) | No record of patient age | Patients without confirmed bacterial diagnosis excluded. |
