# Supplementary material for: A Systematic Review of the Mortality from Untreated Leptospirosis
Source: PLoS Negl Trop Dis. 2015 Jun 25;9(6):e0003866. doi: 10.1371/journal.pntd.0003866 (PMC4482028; doi:10.1371/journal.pntd.0003866)
Supplement: S7 Table — (DOCX) [file pntd.0003866.s014.docx]

### Supplementary Table 7: Median mortality in patient series according to study design

| Patient Series Design | Number of Patient Series | Total Patients | Median Series Mortality (%) (Range %) |
| --- | --- | --- | --- |
| Randomised controlled trial (RCT) | 3 | 71 | 0.0 (0.0 – 0.0) |
| Non-randomised controlled trial (NRCT) | 3 | 68 | 0.0 (0.0 – 9.0) |
| Prospective Case Series | 8 | 605 | 2.7 (0.0– 19.6) |
| Retrospective Case Series | 25 | 2453 | 8.0 (0.0-39.7) |
| Summary of Case Reports | 2 | 193 | 22.4 (20.0-24.8) |
